# Machine Learning-Enhanced Molecular Dynamics of 4,4′-Bipyridine in a Break Junction: A Supramolecular Origin of the High Conductance

**DOI:** 10.1021/acsnanoscienceau.6c00052

**Published:** 2026-05-26

**Authors:** William Bro-Jørgensen, Junfeng Lin, Joseph M. Hamill, Gemma C. Solomon

**Affiliations:** † Department of Chemistry and Nano-Science Center, University of Copenhagen, Universitetsparken 5, DK-2100 Copenhagen Ø, Denmark; ‡ 148492 Institute of Science and Technology Austria, Am Campus 1, 3400 Klosterneuburg, Austria; § 26658 University of Liège, Molecular Systems Research Unit, B6a Sart-Tilman, BE 4000, Liège, Belgium; ∥ NNF Quantum Computing Programme, Niels Bohr Institute, University of Copenhagen, DK-2100 Copenhagen, Denmark

**Keywords:** molecular electronics, single-molecule break junctions, 4,4′-bipyridine, machine learning force fields, molecular dynamics, supramolecular junctions, electron transport

## Abstract

In single-molecule electronics, 4,4′-bipyridine is a well-characterized molecule that serves both as a fundamental testbed for developing experimental methodologies and showcases the complex behaviors expected from molecular electronic components. In break junction experiments, it is generally understood to exhibit a high- and a low-conductance state that are attributed to a tilted and stretched single-molecule configuration. Despite this established view, we suggest a supramolecular origin for these conductance states that has not previously been considered. Our findings indicate that the high- and low-conductance states may arise from the presence of two molecules and one molecule, respectively, challenging the conventional interpretation. Using a state-of-the-art machine learning force field called the neuroevolution potential, we find that the conventional interpretation of a tilted and stretched configuration is inconsistent with our simulations. Instead, our results suggest that the presence of two molecules promotes junction geometries and gold rearrangements that stabilize a high-conductance state whose transmission exceeds twice that of the low-conductance state. Furthermore, we compare this interpretation to the existing literature of diverse experimental data on 4,4′-bipyridine in break junctions and find that it is consistent with the broader body of experimental evidence. Our results highlight the structural complexity of molecular behavior within break junctions, suggesting even more complex dynamics than originally anticipated. In this light, a broader examination of the dynamics of molecules in single-molecule break junctions with machine learning-assisted molecular dynamics is warranted.

## Graphical abstract

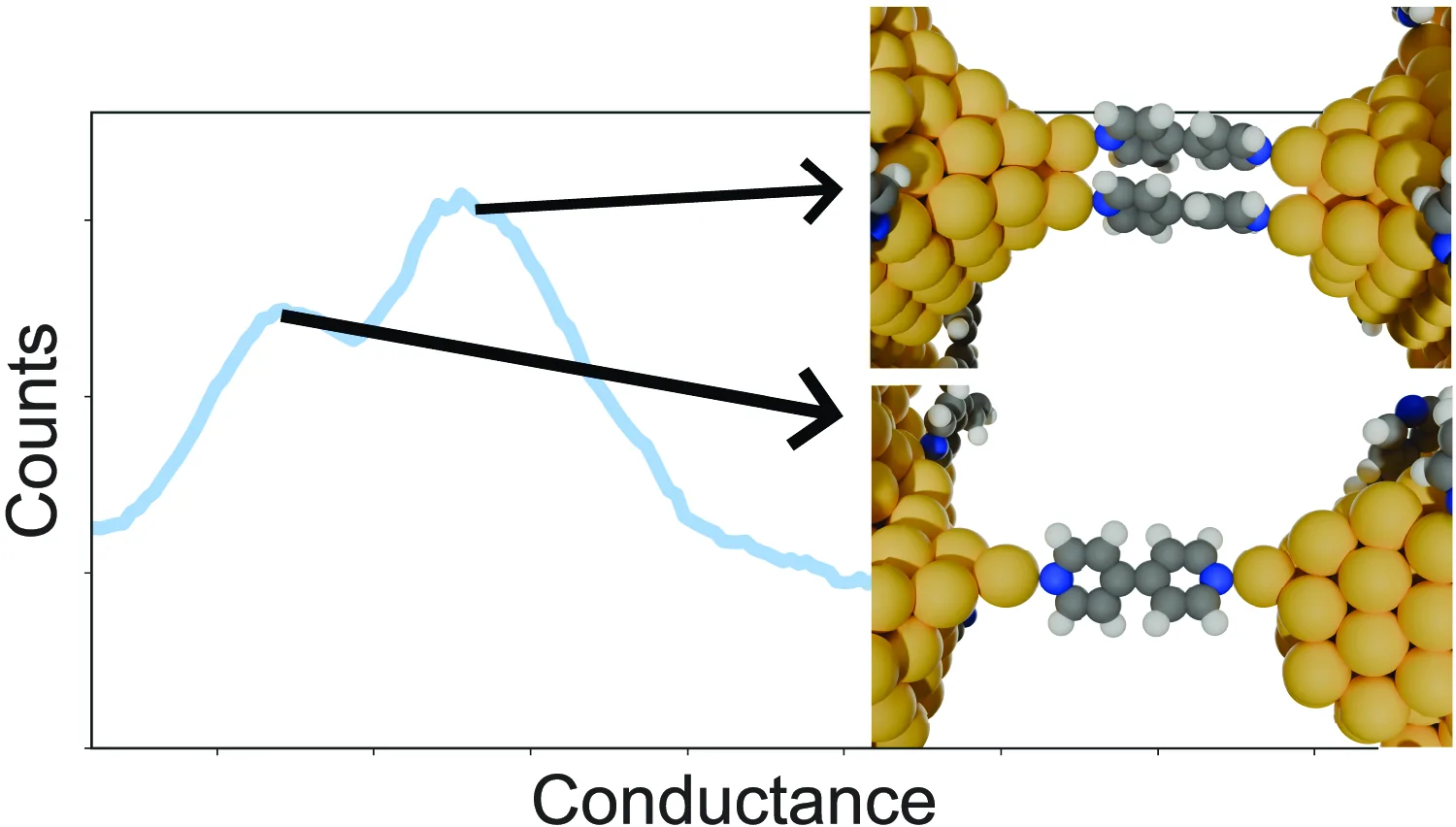

## Introduction

Chemists have a remarkable intuition for inferring intrinsic properties of molecules simply by examining their structure. They can predict where the charge will predominantly reside or anticipate the types of reactions the molecule might undergo. This understanding allows them to identify which modifications to specific functional groups will alter reactivity, and, using only pen and paper, rationalize a reaction mechanism.

However, chemical intuition falters when predicting ensemble properties of the same molecules. For example, it is incredibly challenging to determine, *a priori*, the conditions under which a molecule will crystallize–or whether it will crystallize at all.^1^ The same difficulty applies to predicting solubility, a topic that has been researched for decades.^2,3^

In molecular electronics, it is commonly assumed that we can chemically control the charge transport through single molecules and that our understanding of a single molecular structure informs us about its properties in the junction–that is, we largely ignore dynamics and complex intermolecular effects. By assuming a mostly static junction, we can calculate the transmission of a single or a few junction geometries and still replicate experimental trends. For example, even though room temperature molecular vibrations impact electronic transmission, calculating the transmission at the energy-minimized junction geometry yields qualitatively correct trends.^4^ This assumption is more likely to hold if the properties of the molecule in the junction are similar to the intrinsic properties of molecules, such as reaction mechanisms or charge density, rather than the ensemble properties of crystallization or solubility.

However, even simple molecular systems can exhibit complex transport behavior when placed in a break junction. An example is the relatively simple, rigid 4,4′-bipyridine (44BPY) that was initially expected to show benign transport properties.^5^ It is commonly interpreted that 44BPY adopts two metastable conformations: a tilted and a stretched geometry. The tilted conformation, associated with high conductance, results from enhanced π-system overlap with Au electrodes. The stretched geometry, where the molecule is elongated, leads to lower conductance primarily mediated through the σ-system, facilitated by interacting with the electrodes through the lone pairs on the terminal nitrogen atoms.^6,7^

A growing body of work indicates that Au electrodes are not merely inert contacts: atomic rearrangement accompanies junction formation, and linker chemistry can modulate these rearrangements–for example, thiol linkers promote stronger Au deformation compared with amines.^8^ In addition, the Au electrodes can both catalyze and drive reactions.^9–11^ In parallel, studies increasingly document viable non-Au and nonmetal platforms, e.g., scanning tunneling microscope-based break junction (STM-BJ) with Ag/Cu/Pt/Zn/Ni/Co,^12,13^ or graphene and Si.^14,15^

This complexity reflects the inherently dynamic nature of break junctions. To better interpret single-molecule break junction data, machine learning is increasingly being applied–both to analyze experimental results^16–20^ and to expand the scope of theoretical modeling. In particular, machine learning methods enable more extensive and detailed calculations on single-molecule junctions, offering a more nuanced theoretical understanding of their behavior.^21–25^

With the development of machine learning potentials (MLPs),^26^ it has also become feasible to simulate the time evolution of break junctions involving many molecules, moving beyond the traditional approach based on a few static configurations.

In the following, we use an MLP to simulate a break junction with several 44BPYs to investigate the dynamic nature of these junctions. Specifically, we examine the origin of the high- and low-conductance states. We begin by detailing the generation of the training data for the MLP and evaluating its performance after training. Next, we present transmission calculations performed on snapshots along the molecular dynamics (MD) trajectory. These results demonstrate that two 44BPY molecules between the electrodes can produce two distinct conductance states. To interpret these findings, we decompose the total transmission into contributions from each molecule. This analysis suggests that the enhanced transmission in the supramolecular configuration is not solely due to interactions between the two 44BPY molecules. Instead, the presence of two molecules appears to alter the local gold structure in a way that increases the transmission above the factor two that would be expected if the transmission was a simple sum of two molecules. To further evaluate whether the two conductance states could be explained by a tilted versus stretched configuration, we show the conductance results from a push–pull simulation. In this case, we observe a gradual increase in transmission rather than two distinct states. Finally, we conclude with a discussion of our findings in the context of existing studies on 44BPY, highlighting how our proposed hypothesis aligns with existing experimental observations.

## Methods

We chose the Neuroevolution Potential (NEP) due to its recently demonstrated efficiency in training neural networks to predict materials properties, showing outstanding performance across both crystalline and amorphous materials.^27–32^ For a detailed understanding of the NEP model and its implementation, we refer to the original publications.^32^ The hyperparameters of our trained NEP model are provided in the Supporting Information (SI). We use the same NEP model that we previously trained and applied to investigate the behavior of pure gold junctions.^33^ For completeness, we briefly summarize the training methodology below.

Our model setup involved a system comprising 6 × 6 Au electrodes with either one or two 44BPY molecules. Due to memory constraints during subsequent density functional theory (DFT) calculations, we were unable to use larger electrodes.

We performed DFT calculations using GPAW,^34^ employing the Perdew–Burke–Ernzerhof (PBE) exchange-correlation functional^35^ with Grimme’s D3 dispersion correction^36,37^ and Becke-Johnson (BJ) damping.^38^ Calculations were performed using a double-ζ polarized basis set for all atoms and a ***k***-point sampling scheme of 1 × 1 × 1.

To generate a diverse training set encompassing all relevant conformations, we used an active learning loop based on a force field called Performant implementation of Atomic Cluster Expansion (PACE).^39^ While the performance of the PACE model was worse than the NEP, we reused the training data it generated to train the NEP model due to the efficient exploration of the conformational space of the Au-44BPY-Au junction.

The training set was initially populated with data from two *ab initio* MD simulations–one with a single 44BPY and another with two 44BPYs–each with 6 × 6 Au electrodes. These MD trajectories were performed using GPAW^34^ and ASE^40^ at 700 K. The high temperature was used to accelerate the exploration of conformational space. In these seed runs, we did not pull the electrodes. We employed hydrogen mass repartitioning to facilitate the use of larger time steps, setting the time step at 1 fs.^41^ These seed simulations were run in the microcanonical ensemble (NVE) and with no pulling. We randomly selected 128 structures from the single 44BPY trajectory and 200 from the trajectory with two 44BPYs. Furthermore, we added eight samples from an fcc(111) Au unit cell where we have varied the unit cell parameters to simulate different pressure conditions and capture the bulk behavior of gold.

To generate the training data needed, we employed an active learning loop. For these MD simulations, we used a pulling speed of 2 × 10^–4^ Å/fs, using timesteps of 0.5 fs and a temperature maintained at 300 K using stochastic velocity rescaling with a damping parameter of 100 fs.^42^ This thermostat prevents the junction from heating up due to the mechanical work performed during the stretching process. We imposed periodic boundary conditions in the plane of the electrodes (*x*- and *y*-directions), with nonperiodic, shrink-wrapped boundary conditions along the pulling axis (*z*-direction). The energies of each configuration and the forces on each atom were used to train the NEP model. In all simulations, the velocity of the two outermost layers of gold was set to zero to immobilize them. The active-learning loop consisted of the following steps and a diagram is included in Figure S1:

1. **Initial Training**
  We trained a PACE model using the current training data. This model was used for subsequent MD simulations.
2. **MD Simulation and Data Selection**
  We ran MD trajectories using the PACE model until the trajectory either ran to completion or crashed. During these trajectories, we identified snapshots where the predictions of the model were insufficient, based on a D-optimality criterion from the PACE code.^43^ This criterion assesses the need for model extrapolation for new samples; snapshots requiring significant extrapolation were flagged for further analysis.
3. **Force Calculations**
  For each flagged snapshot, we performed DFT calculations to determine the forces acting on all atoms. These data points – both the forces and the corresponding structures – were then added to our training data set.
4. **Loop Termination**
  We terminated the active learning loop when an entire MD trajectory could run to completion without flagging any new poorly described structures.

The NEP model was ultimately trained directly on energies and forces calculated with DFT from the training data set generated during the active learning.

The electronic transmission was calculated using the nonequilibrium Green’s function (NEGF) approach using the TranSIESTA^44^ module of SIESTA.^45^ For these calculations, we used the PBE exchange-correlation functional with a single-ζ basis set for all atoms and a 1 × 1 × 1 ***k***-point sampling scheme. The self-consistent field mixing parameters were set with a weight of 0.08 and a history of eight steps. All other settings were kept at their default values. No geometry optimization was performed with SIESTA.

For the production MD runs, we modeled a break junction setup by placing two layers of a 10 × 10 fcc(111) Au on either side of a short wire, totaling 717 Au atoms. We included eight 44BPYs evenly distributed around the gold wire. Before starting each MD simulation, the starting geometry was optimized with the NEP model until both the change in total potential energy and the maximum force acting on any atom were below 10^–7^ eV. We used time steps of 0.5 fs. The temperature was again maintained at 300 K in an NVT ensemble using stochastic velocity rescaling with a temperature damping parameter of 100 fs.

We express pulling speeds in Å/fs, aligning with MD simulation conventions, rather than the nm/s typically used in experimental break junction studies. For ease of comparison, we note that our slowest pulling speed is 5 × 10^–8^ Å/fs which corresponds to 10^6^ nm/s–that is, the simulations are pulling 5 orders of magnitude faster than typical experiments.

Our slowest pulling speed is still approximately 5 orders of magnitude faster than typical STM-BJ/mechanically controlled break junction (MCBJ) experiments. This is a computational limitation and should be kept in mind when comparing directly to experiment: faster pulling is expected to, for example, increase plateau lengths, undersample slower electrode and molecular reorganization processes, and reduce the time for a second molecule to diffuse into the junction. All active-learning and production pulling trajectories were performed at 300 K, and the transport analysis is based on snapshots from these 300 K trajectories.

All MD calculations were performed using LAMMPS (28 Mar 2023 - Development).^46^

## Results

In Figure 1A,B, we present both a direct comparison of the forces calculated with DFT and NEP, and a comparison of the DFT-calculated forces with the errors from the NEP model, as suggested in a recent perspective.^47^

**Figure 1:**
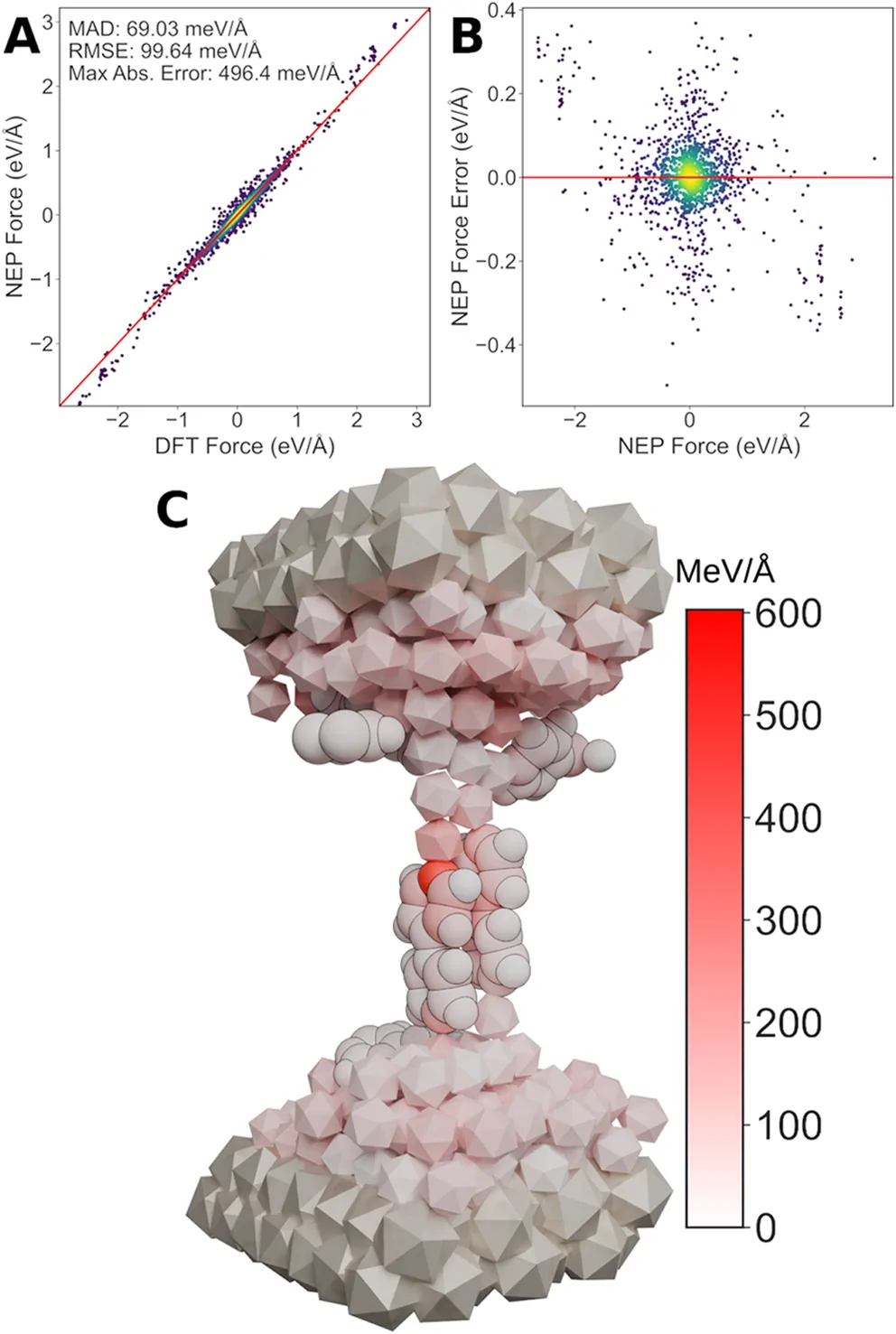
Comparative analysis of atomic forces. (A) Scatter plot comparing the forces on atoms from DFT (*x*-axis) with those predicted by the NEP model (*y*-axis). The mean absolute deviation (MAD), root-mean-square error (RMSE), and the maximum absolute error (Max Abs. Error) are shown in the legend. (B) Scatter plot comparing the forces on atoms from DFT (*x*-axis) with the errors of each force from the NEP model (*y*-axis). The red line is a guide to the eye showing perfect correlation. For visual clarity, the points are colored via kernel density estimation. (C) A visualization of the error on each atom with a gradient going from white to dark red indicating increasing error. To distinguish between atoms in the 44BPYs and the gold, the Au atoms are represented by icosahedrons whereas the atoms in 44BPY are depicted with spheres. The two layers of gold at the top and bottom are frozen in place, meaning their forces are set to 0.

Each point in the data sets represents an individual component (*x*, *y*, or *z*) of a force vector. Overall, the NEP model closely matches the DFT calculations, as indicated by the mean absolute deviation (MAD), root-mean-square error (RMSE), and maximum absolute error detailed in the legend of the plot.

In Figure 1C, we illustrate the force errors for a single structure. The error on each atom uses a color gradient going from white to red, where a deeper red signifies a larger deviation from the DFT-computed force. The error is quantified using the L2-norm, essentially measuring the magnitude of difference between the DFT and NEP forces in all three spatial directions

1 $$\sqrt{\sum_{i=1}^{3}{\left(f_{\mathrm{DFT},i}-f_{\mathrm{NEP},i}\right)}^{2}}$$

Here, the sum runs over each of the three directions (*x*-, *y*-, and *z*-direction), and *f*_DFT_ and *f*_NEP_ are the forces calculated by DFT or predicted with NEP, respectively.

Notably, the error is minimal for almost all atoms, as shown across the structures in Figure 1A,B. The singular high-error atom highlighted in Figure 1C likely represents an isolated outlier rather than a systematic issue and is unlikely to substantially affect the overall accuracy of the MD trajectory predictions.

With the NEP model demonstrating reliable accuracy, we proceed to analyze the electronic transmission of MD trajectories stretched at a rate of 5 × 10^–8^ Å/fs.

Figure 2 shows how the transmission evolves during MD trajectories. In the upper section of the figure, snapshots from key moments in the MD trajectory show the central region of the junction. Here, the red and green boxes highlight traces with two and one bistable conductance states, respectively.

**Figure 2:**
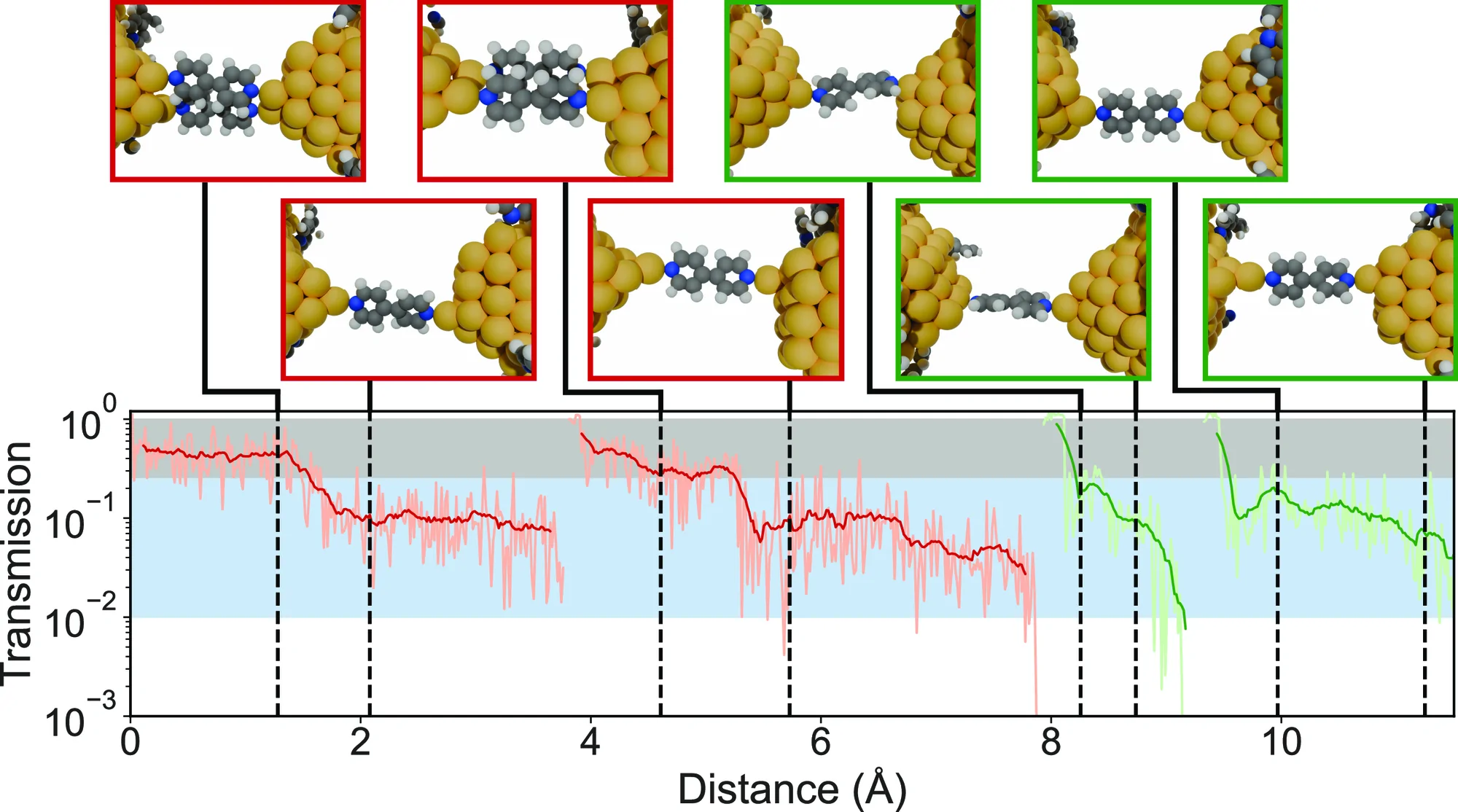
Visualization of the junction evolution and its impact on conductance. Top: Snapshots from selected MD trajectories, stretched at a rate of 5 × 10^–8^ Å/fs, are depicted with outlines color-coded to match their corresponding conductance trace. Bottom: Conductance traces from the MD trajectories, where dashed lines indicate the configurations captured in the top snapshots. To show the general evolution of the transmission, we plot the actual transmission (shown in lighter hues) and its smoothed average (shown in darker hues). Traces are displaced along the *x*-axis for clarity. The gray and blue background in the plot serve as visual aids to delineate the two conductance states. Distance refers to the electrode pulled.

The lower section of the figure displays transmission data for four specific trajectories. The first two, marked in red, demonstrate two bistable conductance states. In contrast, the last two, colored in green, do not clearly show these states. Dashed black lines indicate points in the trajectories corresponding to the snapshots above. To illustrate overall trends, the transmission data are smoothed and shown in darker shades of red and green with the unsmoothed transmission shown in lighter hues.

Only configurations with two molecules appear to show conductance switching substantial enough to produce two distinct states. We refer to this two-molecule configuration as a *supermolecule*.^48,49^ By contrast, single-molecule configurations show only a gradual decline in conductance.

In our simulations, the supermolecules are observed to bind to both electrodes simultaneously, differing from configurations explored in previous studies, such as that of Quek et al.^6^ Their experimental investigations of molecules such as 4-phenylpyridine–a molecule similar to 44BPY, but with only a single nitrogen atom–no distinct molecular peaks were observed in 1D conductance histograms. These experimental results were complemented by theoretical calculations on binding configurations of two 44BPYs where one end binds to the electrode and the other end π-stack with the other molecule. It was concluded that a junction with multiple molecules was inconsistent with the observed bistable conductance states. However, our findings suggest that when molecules can simultaneously bind to both electrodes, stable junctions with two molecules are possible.

Interestingly, the presence of the supermolecule correlates with experimental observations where high-conductance states typically occur at shorter junction lengths compared to low-conductance states. This is likely because the first molecule detaches before the second, implying that the junction is extended further by the time the low-conductance state is observed. This picture also rationalizes why a high conductance plateau is usually followed by a low conductance plateau, rather than the reverse, as once a supermolecule is trapped in the junction, it sets the stage for this transition.

Although based on limited sampling, our simulations indicate that in MD simulations with few 44BPY molecules in the central region, a supermolecule is not observed between the electrodes. This observation suggests a possible concentration-dependence.

We present 1D histograms of the transmission traces in Figure S6. However, due to the limited sample size, we are unable to resolve the double-peak feature in the histogram for either one or two molecules.

To make sure that our results are robust to the quality of our trained NEP model, we further finetune the NEP model for three more rounds using data from pulling trajectories with more 44BPYs and bigger electrodes. The same qualitative distinction between abrupt two-molecule switching and gradual single-molecule evolution is also recovered with the finetuned NEP model on 44BPY pulling simulations. The transmission results are shown in Figures S3 and S4.

To assess the thermodynamic favorability for monomer versus dimer formation and how enthalpic versus entropic factors drive this equilibrium, in “Gibbs Free Energy of Two 4,4′-Bipyridines” in the SI we calculate the relative enthalpic (Δ*H*) and entropic (Δ*S*) contributions to the free-energy difference between the isolated and paired 44BPYs in gas phase. The reaction has Δ*H* = −37.23 kJ/mol indicative of strong binding between two 44BPYs. Despite such strong binding, Δ*G* = 30.45 kJ/mol indicates that this reaction is unlikely to spontaneously occur at room temperature in gas phase. We note that Δ*G* might be overestimated as calculating the entropy contribution for noncovalently bound molecules is challenging.^50,51^ Furthermore, in a junction, one molecule is already constrained between the electrodes, so dimerization incurs a smaller loss of translational and rotational entropy than in gas phase. The entropic penalty is therefore reduced, lowering Δ*G* and making spontaneous binding more plausible.

To further support the results obtained with the NEP model, in “Ab initio MD” in the SI, we present electronic transmission from short *ab initio* MD simulations with one or two 44BPY molecules in the junction. These simulations exhibit the same trend observed with the NEP model: the transmission with two molecules in the junction exceeds twice the transmission of a single molecule.

To investigate the origin of the increased conductance, we attempt to separate the contributions of each molecule to the total conductance. We selected several frames along the first trajectory shown in Figure 2 and computed the transmission in three configurations: with both molecules present, with one molecule removed, and with the other molecule removed. We then compared the transmission of the full two-molecule setup to the sum of the transmissions from the two single-molecule configurations. The results for this single trajectory are shown in Figure 3.

**Figure 3:**
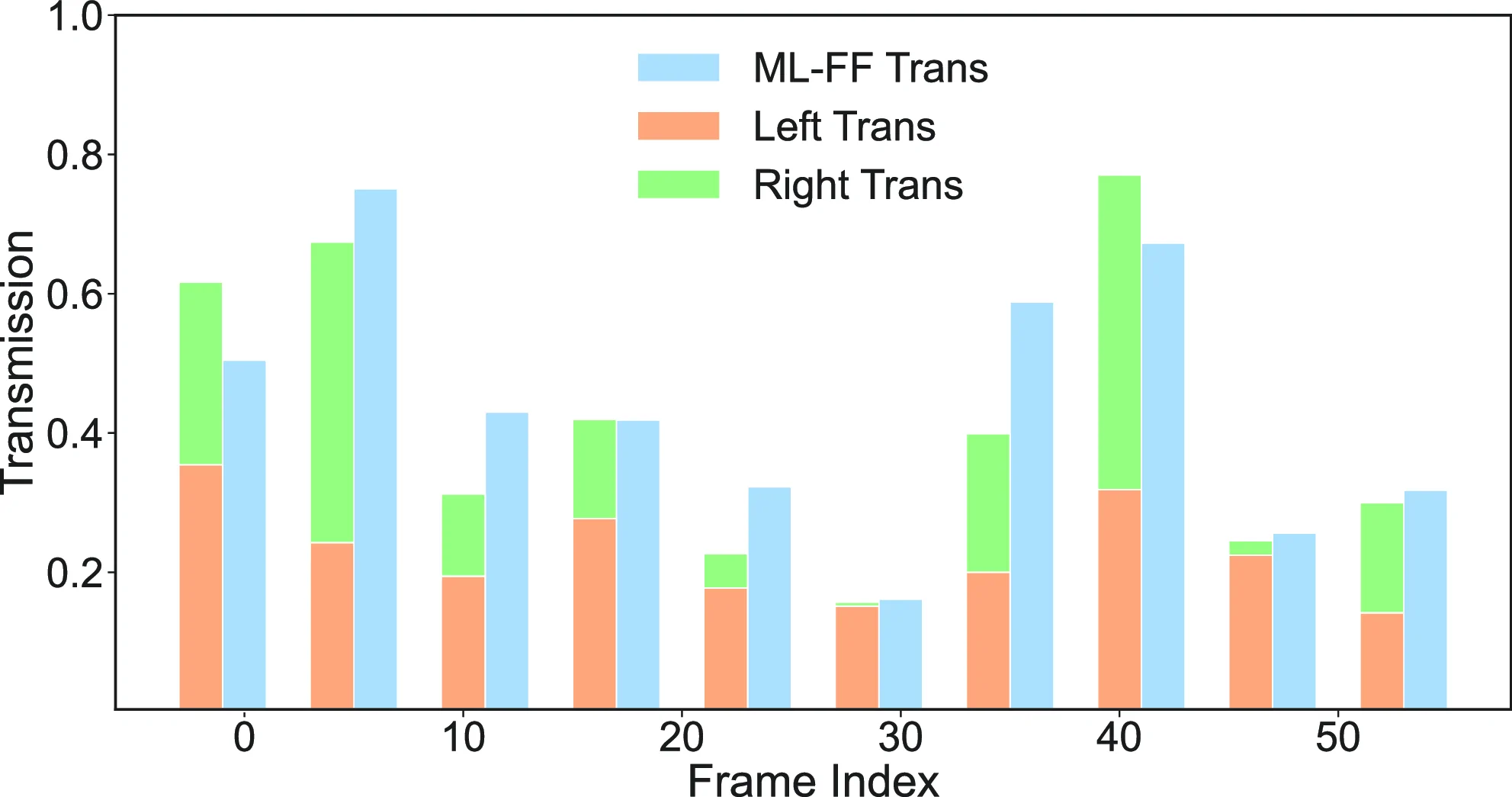
Bar plot of the full double-molecule transmission compared with the sum of the single-molecule transmissions. Green bars are the transmission with only the right molecule (Right Trans), orange bars are the transmission with only the left molecule (Left Trans), and the blue bars are the transmission from the double-molecule junction (ML-FF Trans). The green and orange bars are stacked to show the sum of their transmission.

If the increased transmission were dominated by strong intermolecular interactions, we would expect it to differ substantially from the sum of the individual contributions. Instead, the transmissions are comparable in most cases–the blue bar is approximately equal in height to the stacked green and orange bars. This suggests that the increase in transmission beyond a factor of 2 cannot be explained solely by intermolecular interactions. A tentative hypothesis is that the presence of two molecules modifies the gold structure in a way that enhances transmission.

To explore the extremes of the structural sensitivity in the supermolecule, we probe how junction geometry and electrode coupling influence conductance in “DFT Modelling of Single Configurations” in the SI. Here, we perform DFT calculations on a supermolecule where we remove one or the other 44BPY and either optimize the single-molecule configuration or not.

We find that, depending on the exact location of the Fermi level with respect to the HOMO and LUMO, the ratio between the conductance of the supermolecule and a single molecule can potentially be 30–50 times higher or just slightly more than 2. This illustrates the sensitivity of these calculations to the exact configuration of the molecules and the junction geometry, and the importance of including temperature effects like is done in MD calculations.

In an attempt to isolate the impact of weak π–π coupling on conductance enhancement, we present results from modeling two weakly interacting 44BPY molecules in a junction and their resulting transmission using Hückel theory in “Hückel Modelling of Two 4,4′-Bipyridines” in the SI. This model shows that even weak intermolecular interactions can substantially increase transmission, consistent with the DFT results on individual configurations. However, these idealized configurations are not frequently sampled in the MD simulations, and the additive behavior seen in Figure 3 suggests that intermolecular interactions alone cannot explain the observed enhancement in the MD simulation.

To examine whether the transmission correlates with changes in the Au structure, we plot the transmission against several gold metrics in Figure S5. No single descriptor shows a clear one-to-one correspondence with the transmission. In the two-molecule traces, abrupt transmission changes are accompanied by changes in multiple Au descriptors, whereas the one-molecule traces show fluctuations of similar magnitude in the Au descriptors without comparably abrupt transmission changes. These results suggest that the high-conductance state is not controlled by a single geometric variable, but rather emerges from a more complex junction configuration involving both the molecules and the Au contact structure. This interpretation is consistent with the near-additive behavior in Figure 3 and the absence of discrete switching in the push–pull single-molecule trajectories of Figure 4, arguing against direct intermolecular coupling or a simple tilted-to-stretched coordinate as the main explanation of the high-conductance state.

**Figure 4:**
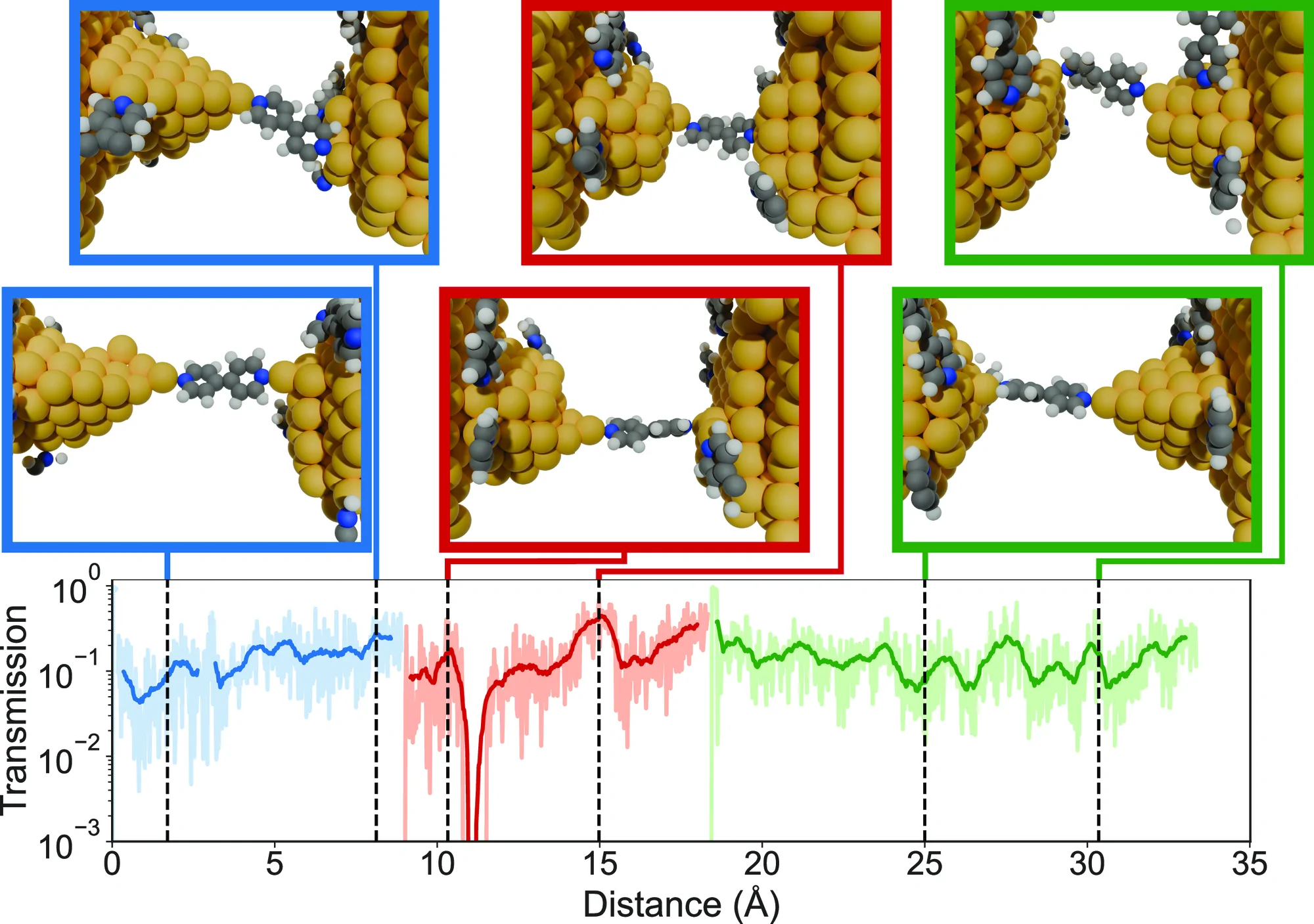
Visualization of the junction evolution and its impact on the conductance. (top) Snapshots from the MD trajectory, stretched at a rate of 10^–7^ Å/fs. (bottom) Conductance traces from the MD trajectory, where dashed lines indicate the configurations captured in the top snapshots. To show the general evolution of the transmission, we plot the actual transmission (shown in lighter hues) and its smoothed average (shown in darker hues). Distance refers to the electrode pushed.

Taken together, these results suggest a complex interplay between geometry, temperature, electrode coupling, and intermolecular interactions. While specific geometries can support very high transmission via intermolecular interactions, such configurations appear to be rare under ambient conditions. The enhancement observed in MD simulations is thus more plausibly attributed to how two molecules collectively restructure or stabilize the junction environment, for example, by altering the gold structure, rather than to direct electronic coupling between them.

In Figure 4, we present the transmission data for the push phase of a push/pull junction simulation. The junction was pulled apart as described earlier, and after the gold breaks, the junction was pushed back together. We plot the transmission (bottom of Figure 4) from the push phase of the trajectory and selected snapshots along the trajectory (top of Figure 4).

As the 44BPY molecule transitions from a fully stretched to a tilted conformation, the transmission does not exhibit a clear distinction between high- and low-conductance states. Instead, we observe a gradual increase in transmission as the junction is compressed, rather than a discrete switch as expected from the tilted/stretched hypothesis.

The discontinuity in the first (blue) trace arises from a few frames where the transmission calculations did not converge. The big dip for the red trace is due to the 44BPY briefly breaking loose of the electrode and then reattaching.

## Discussion

In proposing a supramolecular origin for the high-conductance state observed in 44BPY conductance experiments, we aim to compare this interpretation with existing experimental data on 44BPY. For completeness, we have compiled a table of experimental conductance values for the high- and low-conductance state of 44BPY in Table S4.

Our modeling approach differs from previous work in that the interacting 44BPY molecules bind to different electrode sites.^52,53^ As such, our model captures the behavior of two interacting parallel wires rather than a single parallel pathway. This picture resembles early theoretical studies on conductance through parallel molecular wires.^54^ It also allows for the observed transmission ratios exceeding 4 in our calculations.

Other dimer-forming molecules, such as 4,4″-diamino-*p*-terphenyl and 2,7-diamino fluorene, exhibit a similar pattern, with conductance ratios between high and low states reaching values around 10.^55^ Likewise, in systems where two phenyl moieties are mechanically forced into π-stacked configurations, an increase in conductance is also observed.^56^ In experiments comparing paracyclophane with a para-substituted benzene, the high-to-low conductance ratio was found to be 2.8, with the cyclophane corresponding to the high state and the benzene to the low state.^52^ This experiment further supports the idea that the ratio between two molecules could be higher than 2.

Previously, the possibility that multiple 44BPY molecules might be the origin of either conductance state within a single junction was considered but ultimately dismissed. Control experiments with molecules similar to 44BPY, such as 4-phenylpyridine (containing only one nitrogen) and 2,4′-bipyridine (with a nitrogen in the meta-position instead of the para-position), showed no molecular peaks in 1D- and 2D-histograms, ruling out both strongly asymmetric junctions, where only one side binds to the electrodes, and junctions with a π-stacked molecular conformation.^6^ Additionally, the ratio of high to low conductance peaks, which exceeds 2, has been viewed as further evidence of the presence of a single molecule in the junction. If two noninteracting molecules were measured in the junction, a ratio of approximately 2 would have been expected. Experimentally, this ratio has been observed to vary between 3 and 5.^6,57^ See also Table S4 for a table of experimental values of the high- and low-conductance peaks.

We acknowledge a previous study that considered the presence of two 44BPYs in a single junction as a possible explanation for the high-conductance peak.^58^ However, the main focus of that study was on effective electrochemical gating of single-molecule junctions. As a result, the mechanisms by which two 44BPYs could lead to a high-conductance state were not explored in detail.

Evidence for supramolecular interactions involving 44BPY and related molecules has been reported in various experimental contexts, supporting the plausibility of such configurations in break junctions. For instance, π-stacking has been observed in experiments on monolayers of 44BPY on Au(111) substrates, though conductance was not measured.^59,60^ 44BPY is also known to form supermolecule-conformations between metal centers in coordination polymers.^61^ Supermolecule-conformations of other molecules have previously been invoked to explain multiple observed conductance states from break junction experiments.^55,62–67^ These supermolecules are either formed through π-stacking or other noncovalent bonding mechanisms.

We next revisit experimental observations that have been interpreted in support of the tilted/stretched hypothesis and show how this evidence is also consistent with the supermolecule hypothesis.

1. **Concentration Dependent Experiments**
  Concentration-dependent experiments on 44BPY have previously been reported.^68,69^ In the study by Kotiuga et al.,^68^ conductance measurements were performed in a 44BPY solution at a concentration of 10^–5^ M, with solvent evaporation over 5 h, leading to an increase in 44BPY concentration. Although the data in their SI are difficult to quantify precisely, it appears that the high-conductance peak increases more than the low-conductance peak as the concentration rises.
  A recent study by Long et al.^69^ directly measured the concentration dependency of 44BPY break junctions. They observed that the high-conductance peak diminishes more rapidly than the low-conductance peak with decreasing concentration. Based on this result and further analysis, they conclude that the two conductance states remain consistent with the tilted/stretched configuration. They attributed the concentration dependency to a crowding effect, where at higher concentrations, more molecules near the bridging molecule force it to adopt a more tilted configuration. As the concentration decreases, the crowding diminishes, allowing the 44BPY to adopt a more stretched configuration, thus explaining the observed concentration dependency.
  We suggest that the supermolecule hypothesis provides a more consistent explanation for the observed concentration dependence and the MD simulations than the crowding-based model.
2. **van der Waals Interactions**
  Aradhya et al.^7^ used an atomic force microscope (AFM) to probe the rupture force and stiffness of single-molecule junctions with 44BPY. They observed an average rupture force for the high conductance state that was higher than expected for Au–Au bond rupture. Likewise, the average stiffness for the high conductance state was observed to be higher than that of single Au atom contacts. Both results can be understood by considering a supermolecule configuration. With two molecules in the junction, it is expected that both the average rupture force and the average stiffness will be higher.
3. **Force Spectroscopy**
  Force spectroscopy measurements on 44BPY, reported by Magyarkuti et al.,^55^ showed no direct correlation between the applied force and the conductance. This observation was interpreted as evidence against dimer formation. That conclusion is reasonable for a geometry in which one molecule is attached to each electrode and elongation progressively weakens the intermolecular contact. However, it does not strictly exclude the alternative supermolecule configuration in which both 44BPYs interact with both electrodes. In such a two-molecule configuration, the measured force could be governed mainly by electrode-molecule anchoring, while the conductance is controlled by, for example, contact geometry, leading to little or no force-conductance correlation. The original study did not explicitly consider this possibility.
4. **Push–Pull**
  Quek et al.^6^ performed push–pull break junction experiments with 44BPY and observed that changing the junction gap by only 2 Å could switch between the high and low conductance state without breaking the junction. Larger or smaller push/pull cycles led to unstable junctions or no observed switching, respectively. As explained in the previous section, we observed the supermolecule configuration to always precede the single-molecule configuration. From that observation, we conclude that the supermolecule is only able to fit in relatively small junction gaps. At larger junction gaps, the second molecule is unable to bind efficiently to both sides of the electrodes. Therefore, when the push/pull cycle is 2 Å, the junction gap switches between a gap size that can accommodate the supermolecule and a gap size that cannot.
5. **Different Electrodes**
  In their study using an scanning tunneling microscopy break junction (STM-BJ) setup with silver electrodes, Adak et al.^70^ observed only a single peak in the 1D-conductance histograms for 44BPY. Prior research by Kim et al.^71^ indicates that silver electrodes sustain longer elongation than Au electrodes. Consequently, if the formed nanogap is too large, the second molecule will be unable to bind, preventing the appearance of the high-conductance state and only a single peak is seen in the conductance histograms.
6. **Conductance at 4.2 K**
  MCBJ experiments conducted on 44BPY at cryogenic temperatures predominantly yield conductance traces with the low-conductance peak.^72,73^ In experiments using STM-BJ setups, other groups have observed that at cryogenic temperatures, gold tips are corrugated and retain a sharp form, whereas at room temperature, they melt into a smoother configuration.^74^ This phenomenon explains the formation of larger nanogaps between the junctions at lower temperatures, which then cannot support the supermolecule configuration of 44BPY associated with the high-conductance state.
  As further evidence of this explanation, analysis of the length distribution of 1G_0_ plateaus from traces showing only the low-conductance state and traces showing both states offers intriguing insight.^72^ Samples displaying solely the low-conductance peak pull out more gold atoms compared to those also showing a high-conductance peak. Pulling out more gold results in a physical constraint: as a chain of gold has been pulled out, there may be no suitable gold binding site left for the second 44BPY, hence only the low-conductance peak is observed. This situation is corroborated by the snapshots with two 44BPYs shown in Figure 2 where each 44BPY binds to a distinct gold site.
7. **Voltage-Controlled Switching**
  A study by Mezei et al.^73^ found that the switching rate between the high- and low-conductance states increased with higher applied voltage. The researchers compared these experimental observations with various models and concluded that a heuristic model, which relies on the exponential energy dependence of the molecule’s transition probability, provided the best explanation.
  Invoking the idea of a supermolecule, one possible explanation is as follows: In “Energy of Two 4,4′- Bipyridines under an Electrostatic Field” in the SI, we show results for the total energy of two 44BPYs in gas phase as they are moved further apart. We show an energy profile for three different situations: without an electrostatic field, a field with a strength of 2.57 V/nm, and a field with a strength of 5.14 V/nm. These calculations show that increasing the field strength lowers the energy of both the supermolecule configuration and the two separated molecules. This decrease in energy allows the high-energy state, which is less populated at the experimental temperature, to become more accessible. Consequently, as the field strength increases, the likelihood of the molecule switching between the two states also increases. This finding supports the heuristic model of the original study that suggested a reduction in energy can exponentially increase the switching rates between the two states.
  Similarly, a study by Tang et al.^75^ found that increasing the strength of the applied electrostatic field in an STM-BJ increases the amount of traces with terphenyl stacked dimers. This experimental result further supports the hypothesis that modulating the applied bias across break junctions will affect the formation of supermolecules, including 44BPY.
8. **Correlation Analysis**
  Makk et al.^76^ analyzed 2D-correlation histograms for 44BPY obtained from STM-BJ measurements at room temperature and ambient conditions. They observed a negative correlation between the high-conductance and the low-conductance state, concluding that this anticorrelation was due to the lengths of the plateaus in the two states. Specifically, a shorter-than-average plateau in one state typically led to a longer-than-average plateau in the other state. To explain this under the supermolecule hypothesis, consider that the junction can only be pulled a certain extent–irrespective of the number of molecules caught in the junction. If the junction has already been pulled for a prolonged period with a supermolecule and then the first molecule breaks away, the junction cannot sustain the second molecule for long. And vice versa, if the first molecule detaches early, the junction will be able to sustain the second molecule for a long time. Thus, an anticorrelation will be measured.
9. **Thermopower**
  Several studies have measured the thermopower of 44BPY, all agreeing that transmission is mediated by the LUMO, and consistent with the predictions for a supermolecule configuration (for example, see Figures S9B, S13, or S10).^77–79^ However, these studies present conflicting experimental data regarding which of the high- or low-conductance state has the lowest thermopower. The first study predicts that the high-conductance state has the lowest thermopower, whereas the latter two studies predict that it is the low-conductance state that has the lowest thermopower. Moreover, as pointed out by Vavrek et al.,^79^ the estimated thermopower for both conductance states are statistically indistinguishable within the experimental error across all three studies. Consequently, it is challenging to conclude in favor of either the tilted/stretched hypothesis or the supermolecule hypothesis, although both hypotheses align with the experimental thermopower data.
10. **Energy Level Alignment**
  In the study by Kim et al.,^77^ an estimation of the energy level alignment and coupling strength in 44BPY junctions was performed using an STM-BJ experimental setup. It was found that the coupling strength in the high-conductance state was higher. This observation is consistent with a supermolecule, which would be expected to couple more strongly to the electrodes.
  Additionally, the electronic molecular energy level alignment was also found to be higher for the high-conductance state, indicating that the LUMO for the high-conductance state is positioned further away from the Fermi level than the low-conductance state. This result contradicts what would be predicted with a supermolecule configuration (for example, see Figures S9B or S10).
  Despite this apparent inconsistency with the supermolecule configuration, we note two things: first, as previously remarked, the estimated thermopower for both conductance states are statistically identical within the experimental error across three studies. If the thermopower for both states is indeed the same, then the high-conductance state will lie *closer* to the Fermi level–that is, in agreement with the supermolecule configuration. Second, if we set aside the experimental error, only the study by Kim et al.^77^ finds the high conductance state to have the lowest thermopower. The other two studies predict the reverse ordering. This discrepancy in the ordering of the thermopower is important for estimating the energy level alignment and if the ordering is reversed, it is likely that the energy level alignment also reverses.
11. **Prior Theoretical Calculations**
  Previous papers discuss theoretical transmission calculations to explain the observed behavior of 44BPY. Specifically, Quek et al.^6^ demonstrated that as 44BPY stretches across the junction, the conductance decreases gradually. This gradual decrease is observed across multiple structures, suggesting a continuous, rather than a discrete, drop in conductance.
  Similarly, Hong et al.^80^ performed electronic transmission calculations of simulated stretching trajectories of tolane molecules with pyridine linkers. While the experimental data suggested at least two plateaus, the theoretical calculations only showed a smooth transition from a high conductance state to a low conductance state. In a subsequent paper, the same group concluded that break junction experiments on tolane molecules with pyridine linkers might be measuring a supermolecule.^81^ This conclusion was based on breaking force and conductance measurements, as well as calculations of those same properties at every elongation step.
  These findings imply that the smooth changes in conductance observed in theoretical calculations differ from the discrete, step-like transitions expected if the tilted geometry was the origin of the two conductance states seen in experiments.
12. **Flicker Noise**
  A study by Balogh et al.^82^ explored the flicker-noise characteristics of 44BPY. However, it is challenging to use this data to refute either hypothesis. Both hypotheses suggest that the observed flicker noise would result from through-space conductance when examining the high-conductance peak. In the supermolecule picture, each 44BPY remains bound to the electrodes, but their close proximity leads to fluctuations in their intermolecular coupling. This fluctuation leads to fluctuations in the conductance. In contrast, the tilted-geometry hypothesis attributes the through-space component to interactions between the π-system of a single 44BPY and the gold electrodes. In both cases, the low-conductance peak is attributed to through-bond conductance from a single 44BPY molecule stretched across the junction. Because both hypotheses are consistent with the reported flicker-noise behavior, these data do not discriminate between them.
13. **UV/vis Irradiation**
  Tan et al.^83^ performed ultraviolet (UV) radiation of 44BPY in an STM-BJ experimental setup. They observed a continuous decrease in conductance upon UV irradiation at 365 nm. They conclude that this decrease in conductance is due to a *n* → π transition. The findings from this experiment do not exclude either the supermolecule or the tilted/stretched hypothesis, as both could potentially accommodate the observed changes in electronic behavior induced by UV exposure.

## Conclusion

In summary, this study presents a supramolecular explanation for the high-conductance state observed for 44BPY in single-molecule break junction experiments. Using a state-of-the-art machine learning force field trained on DFT calculations, we performed MD simulations of 44BPY in Au-junctions. Our results indicate that a supermolecule conformation gives rise to a high conductance state that switches to a low conductance state when one molecule diffuses away.

To test this hypothesis, we decomposed the total transmission into contributions from each molecule by comparing the full two-molecule configuration with the sum of single-molecule configurations. The results show that the full two-molecule transmission is comparable to the sum of the corresponding single-molecule transmissions, indicating that intermolecular interactions are unlikely to be the primary cause of the enhanced conductance. Instead, we conclude that the presence of two molecules perhaps modifies the local gold structure in a way that increases transmission.

We also presented results of a push–pull simulation alongside the calculated conductance during this simulation. The transmission increases gradually going from a stretched to a tilted configuration. There is no binary switch from a low-conductance state to a high-conductance state as predicted from the tilted/stretched hypothesis.

We also include simulations from a further finetuned NEP model that shows the same qualitative trends: only traces with two molecules show an abrupt change in transmission during junction evolution.

We compared our proposed mechanism with previous studies on 44BPY in break junctions and found that it provides a consistent explanation for the majority of available experimental data.

These findings challenge a widely accepted interpretation in single-molecule break junction research and demonstrate the utility of machine learning-accelerated MD simulations in advancing our understanding of molecular junctions. This new perspective on 44BPY behavior also opens the door to targeted experiments that could distinguish between the supramolecular and tilted/stretched hypotheses. We hope experimental groups can use this proposed origin of the two conductance states to design experiments that can distinguish between the two hypotheses–that is, is it a supermolecule or is it a tilted/stretched configuration? For example, carefully performed concentration dependent experiments with both an STM-BJ and an MCBJ, or where the concentration of 44BPY is diluted with another molecule that is expected to prevent the formation of the supermolecule could provide valuable insight.

Finally, we note that our simulated pulling speeds are orders of magnitude faster than experimental ones, and it has been established that pulling speed affects breaking kinetics.^84^ As we have recently shown,^33^ slower pulling speeds produce narrower junction gaps due to shorter gold chains being drawn out, which could increase the likelihood of 44BPY molecules remaining bound. Moreover, our fast pulling rate likely undersamples slow electrode reorganization and allows less time for a second molecule to diffuse into the junction. Both effects suggest that supermolecule configurations may be more prevalent under experimental conditions than our simulations indicate.

We anticipate that both our supramolecular model and the use of machine learning-based MD simulations will stimulate further exploration into the structural and electronic dynamics of break junctions.

## Supplementary Material



## Data Availability

Input and output files for the MD trajectories are available in our ioChem-BD repository^85^ and can be accessed here: 10.19061/iochem-bd-9-14. Raw data for all MD trajectories are available here: 10.17894/ucph.78aa9ec7-d132-447f-aaf0-8e6e5d59d8ea

## Notes

Hyperparameters for the NEP model; diagram of active learning procedure; SOAP-based selection of active-learning structures; transmission results from SOAP-based active-learning; correlation with gold metrics; Gibbs free energy of two 4,4′-bipyridines; 1D-histogram of transmission traces; ab initio MD; Hückel modeling of two 4,4′-bipyridines; Hückel matrix of a single 4,4′-bipyridine; energy of two 4,4′-bipyridines under an electrostatic field; DFT modeling of single configurations; experimental high- and low-conductance values (PDF)
