# Supplementary material for: Machine Learning-Enhanced Molecular Dynamics of 4,4′-Bipyridine in a Break Junction: A Supramolecular Origin of the High Conductance
Source: ACS Nanosci Au. 2026 May 26;6(4):662–73. doi: 10.1021/acsnanoscienceau.6c00052 (PMC13495496; doi:10.1021/acsnanoscienceau.6c00052)
Supplement: Supplementary file 1 [file ng6c00052_si_001.pdf]

Supporting Information: Machine Learning-Enhanced Molecular Dynamics of 4,4'-Bipyridine in a Break Junction: A Supramolecular Origin of the High Conductance

William Bro-Jørgensen,[†] Junfeng Lin,[‡] Joseph M. Hamill,[†,¶] and Gemma C. Solomon[*,†,§]

†*Department of Chemistry and Nano-Science Center, University of Copenhagen, Universitetsparken 5, DK-2100, Copenhagen Ø, Denmark*

‡*Institute of Science and Technology Austria, Am Campus 1, 3400 Klosterneuburg, Austria*

¶*University of Liège, Molecular Systems Research Unit, B6a Sart-Tilman, BE 4000, Liège, Belgium*

§*NNF Quantum Computing Programme, Niels Bohr Institute, University of Copenhagen, DK-2100 Copenhagen, Denmark*

E-mail: gsolomon@chem.ku.dk

Hyperparameters for the NEP model

We show the hyperparameters for the NEP model in Table S1. All other settings were kept at their default values. The hyperparameters are defined as follows: r_c^R and r_c^A is the radial and angular cutoffs, respectively; N_{bas}^R and N_{bas}^A are number of radial and angular basis functions, respectively; n_{max}^R and n_{max}^A are the size of the radial and angular basis, respectively; l_{3b}^{max}-l_{4b}^{max}-l_{5b}^{max} is the expansion order for angular terms; N_{neu} is the number of

neurons in the hidden layer; Batch size is the batch size used when training the model; and N_{gen} is the number of generations used by the separable natural evolution strategy used to train the model.[S1]

Table S1: Hyperparameters.

Hyperparameter	Value
NEP Version	4
r_c^R (Å)	8
r_c^A (Å)	4
N_{bas}^R	12
N_{bas}^A	12
n_{max}^R	10
n_{max}^A	6
l_{3b}^{max}-l_{4b}^{max}-l_{5b}^{max}	4-2-1
N_{neu}	40
Batch size	1024
N_{gen}	4e6

Diagram of active learning procedure

Figure S1 illustrates the workflow for the PACE-driven active learning.

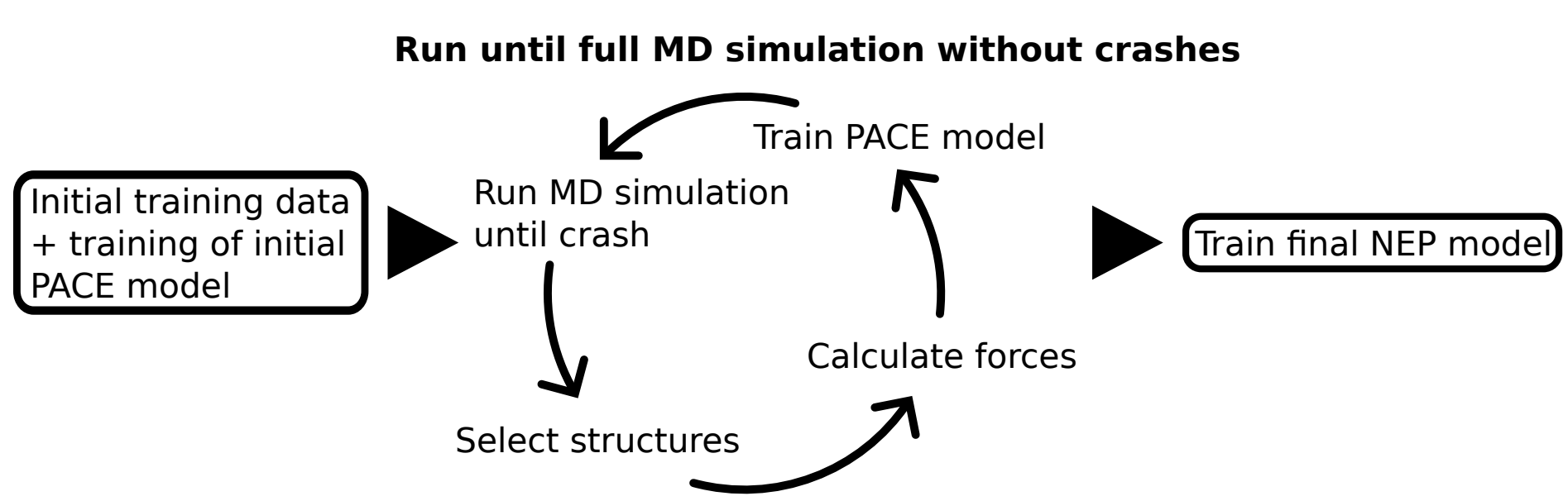

Figure S1: Illustration of the workflow to train the NEP model. Reproduced from ref S2. Available under a CC-BY 4.0 license. Copyright 2025 The Authors. Published by American Chemical Society.

SOAP-based selection of active-learning structures

The Smooth Overlap of Atomic Positions (SOAP) selector was applied after candidate configurations had been generated during the three active-learning rounds. To remove near-

duplicate snapshots, we followed the descriptor-based similarity selector introduced by Zhang et al.[S3] For each candidate structure, we computed a normalized SOAP vector $\mathbf{p}_0$ using $r_{\text{cut}} = 8.0$ Å, $n_{\text{max}} = 8$, $l_{\text{max}} = 6$, $\sigma = 0.5$, non-periodic boundary conditions, and inner averaging. The SOAP descriptors were calculated using `DScribe`.[S4,S5] The similarity to the existing training set was then evaluated as

$$K_i = |\mathbf{p}_0 \cdot \mathbf{p}_i|^{\zeta}, \tag{1}$$

Here, $\mathbf{p}_0$ is the normalized SOAP descriptor of the candidate structure, $\mathbf{p}_i$ is the normalized SOAP descriptor of structure i already in the training set, K_i is the corresponding similarity measure, and ζ is a sensitivity exponent, set to 2 in this work. A candidate was retained only if $\max K_i < k_T$ where k_T is a similarity threshold. Accepted structures were inserted greedily, such that subsequent frames were evaluated against these newly added structures.

To reduce the contribution from the frozen bulk Au atoms, the SOAP vectors were evaluated only for the central part of the junction after removing Au electrodes from both ends. The full, unstripped configurations were retained in the final training set.

We use a larger junction structure than in the main manuscript. The gold electrodes are 10×10 instead of 6×6 and we include 6 44BPYs. This way, we sample a much greater variety of configurations within each frame.

In round 1, all frames from four representative trajectories were screened with $k_T = 0.9995$, which added 24 structures. In round 2, five trajectories were sampled with $k_T = 0.999$, which added 17 structures. In round 3, seven trajectories were sampled with $k_T = 0.9995$, which added 149 structures. These structures were added to the pre-existing training set of 2026 configurations, yielding a final training set of 2216 structures. The skipped structures were shuffled, and 150 were written out as a test set for each round.

In round 1 and 2, the trajectories were pulled with a pulling speed of 5×10^{-7} Å/fs and in round 3, we used a pulling speed of 5×10^{-8} Å/fs.

Figure S2 compares the DFT and NEP forces for the final test set obtained after the three additional active-learning rounds. Relative to the original model, the finetuned NEP yields lower MAD and RMSE, although the maximum absolute error is larger because of a small number of outliers, likely a single point. That outlier is omitted from the plot for clarity because its force is around -15 eV/Å. Figure S2C shows the atom-resolved force errors for one representative test structure. The errors are small for most atoms and somewhat larger on the 44BPY molecules than on Au, but remain lower overall than in Figure 1.

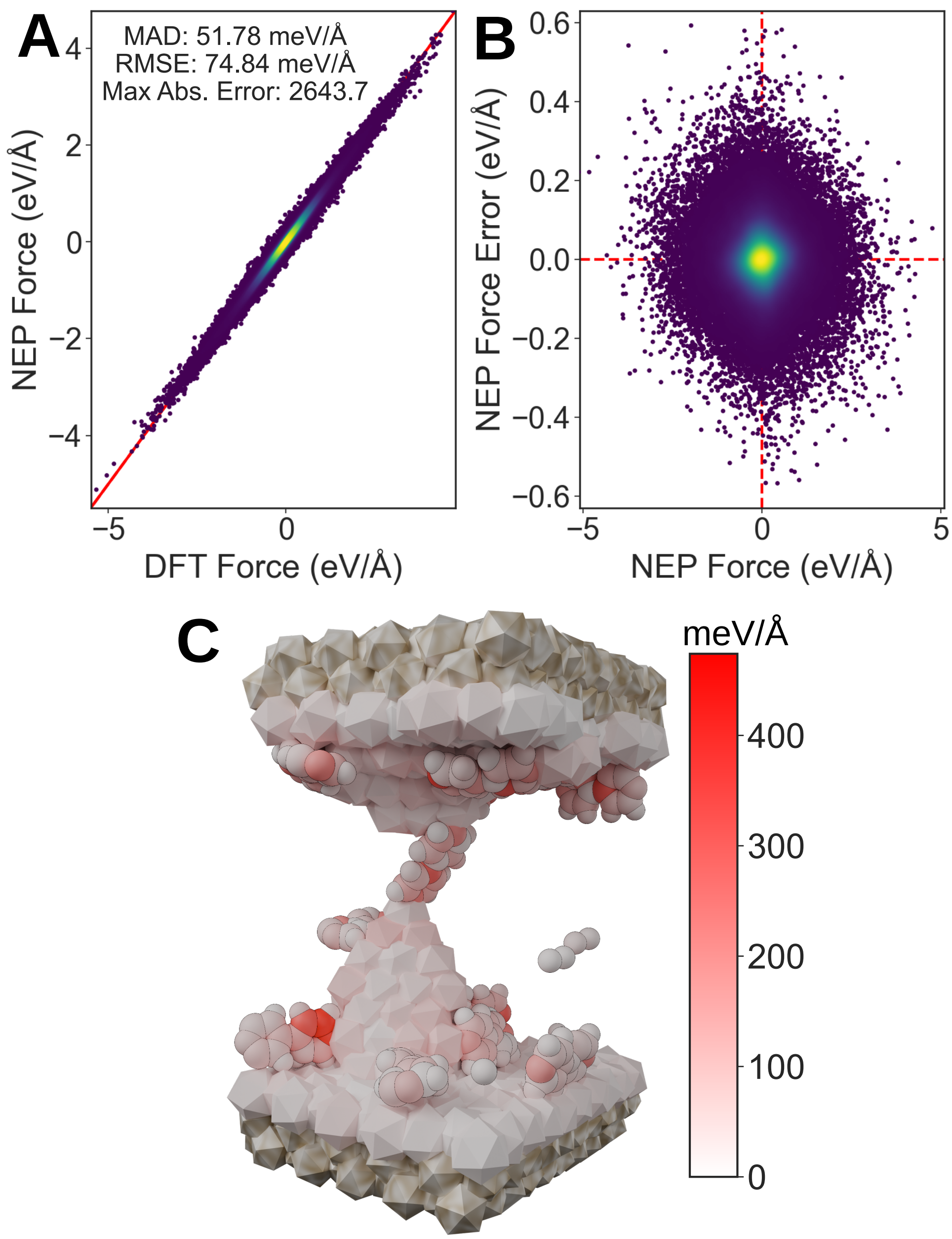

Figure S2: Comparative analysis of atomic forces. (A) Scatter plot comparing the forces on atoms from DFT (x-axis) with those predicted by the NEP model (y-axis). The mean absolute deviation (MAD), root mean square error (RMSE), and the maximum absolute error (Max Abs. Error) is shown in the legend. (B) Scatter plot comparing the forces on atoms from DFT (x-axis) with the errors of each force from the NEP model (y-axis). The red lines are a guide to the eye showing perfect correlation. For visual clarity, the points are colored via kernel density estimation. (C) A visualization of the error on each atom with a gradient going from white to dark red indicating increasing error. To distinguish between atoms in the 44BPYs and the gold, Au atoms are represented by icosahedrons whereas the atoms in 44BPY are depicted with spheres. The two layers of gold at the top and bottom are frozen in place, meaning their forces are set to 0.

Transmission results from SOAP-based active-learning

Figure S3 shows the transmission from selected trajectories using the NEP model that was finetuned using the methodology described in "SOAP-based selection of active-learning structures". The blue and red traces are representative supermolecule trajectories illustrating both the higher-than-2-ratio transmission and the abrupt change in transmission when the second molecule diffuses away. By contrast, the green and magenta traces are single-molecule trajectories in which 44BPY slides over the gold pyramids before becoming fully stretched between the electrodes. In these cases, the transmission changes more gradually and no discrete switch is observed, in line with the conclusion that the bistable behavior is associated with the supramolecular configuration rather than a tilted-to-stretched transition of a single molecule.

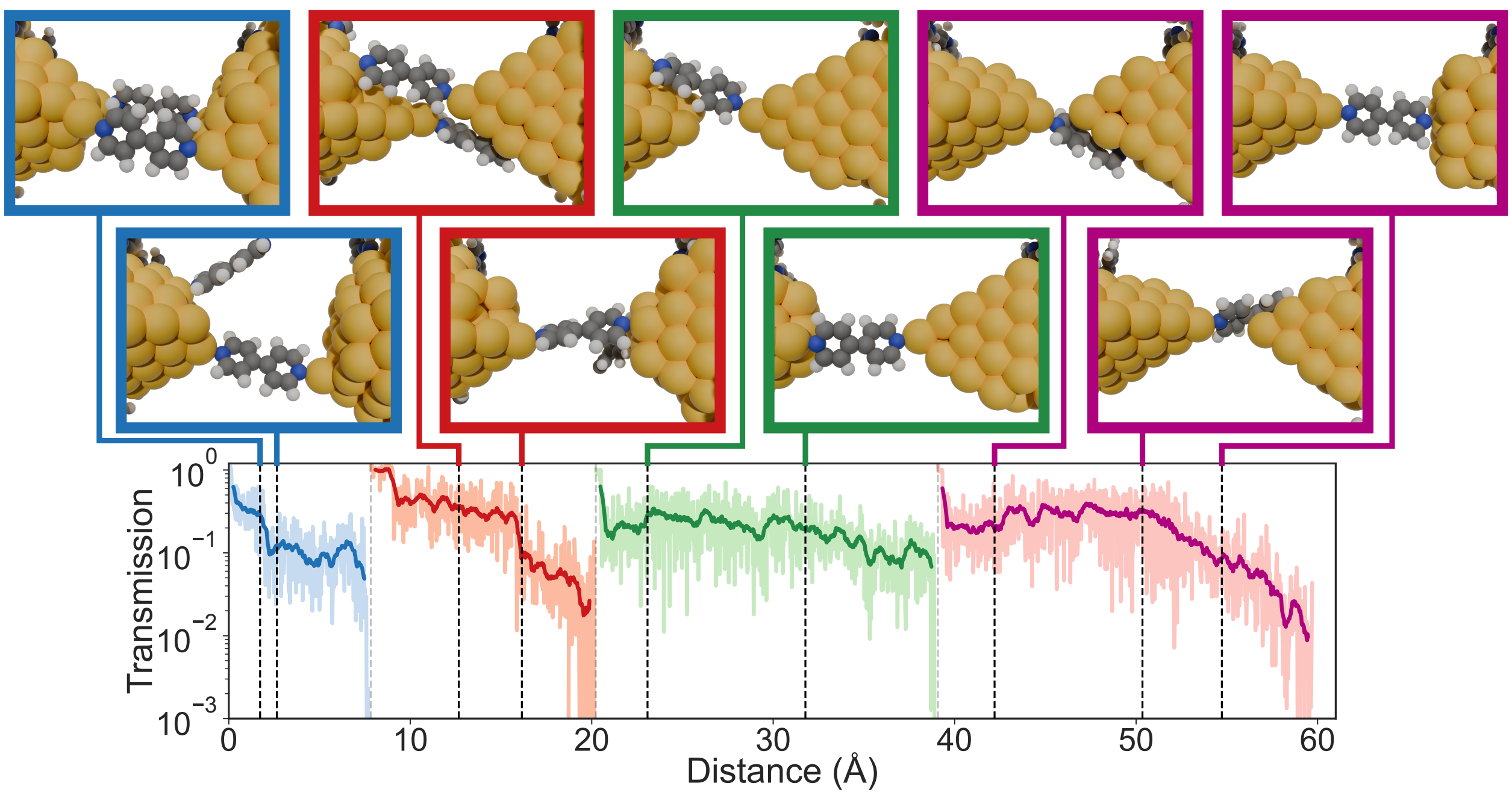

Figure S3: Visualization of the junction evolution and its impact on the conductance. (top) Snapshots from the MD trajectory, stretched at a rate of 5×10^{-7} Å/fs. (bottom) Transmission traces from the MD trajectory, where dashed lines indicate the configurations captured in the top snapshots. To show the general evolution of the transmission, we plot the actual transmission (shown in lighter hues) and its smoothed average (shown in darker hues).

Figure S4 shows less clear-cut examples of both trajectories that briefly contain two

molecules in the junction (blue, red, and magenta traces) and trajectories in which the molecules slide across the gold pyramids (green, purple, and orange traces). For the two-molecule cases, the conductance changes are less sharp than in Figure S3 and Figure 2, although step-like drops are still visible in the red and magenta traces. These examples suggest that the presence of two molecules in the gap is not sufficient to produce a pronounced high-conductance state; rather, both molecules likely need to simultaneously form effective contacts to the electrodes. By contrast, the traces associated with sliding over the pyramids remain comparatively continuous and do not show similarly abrupt switching.

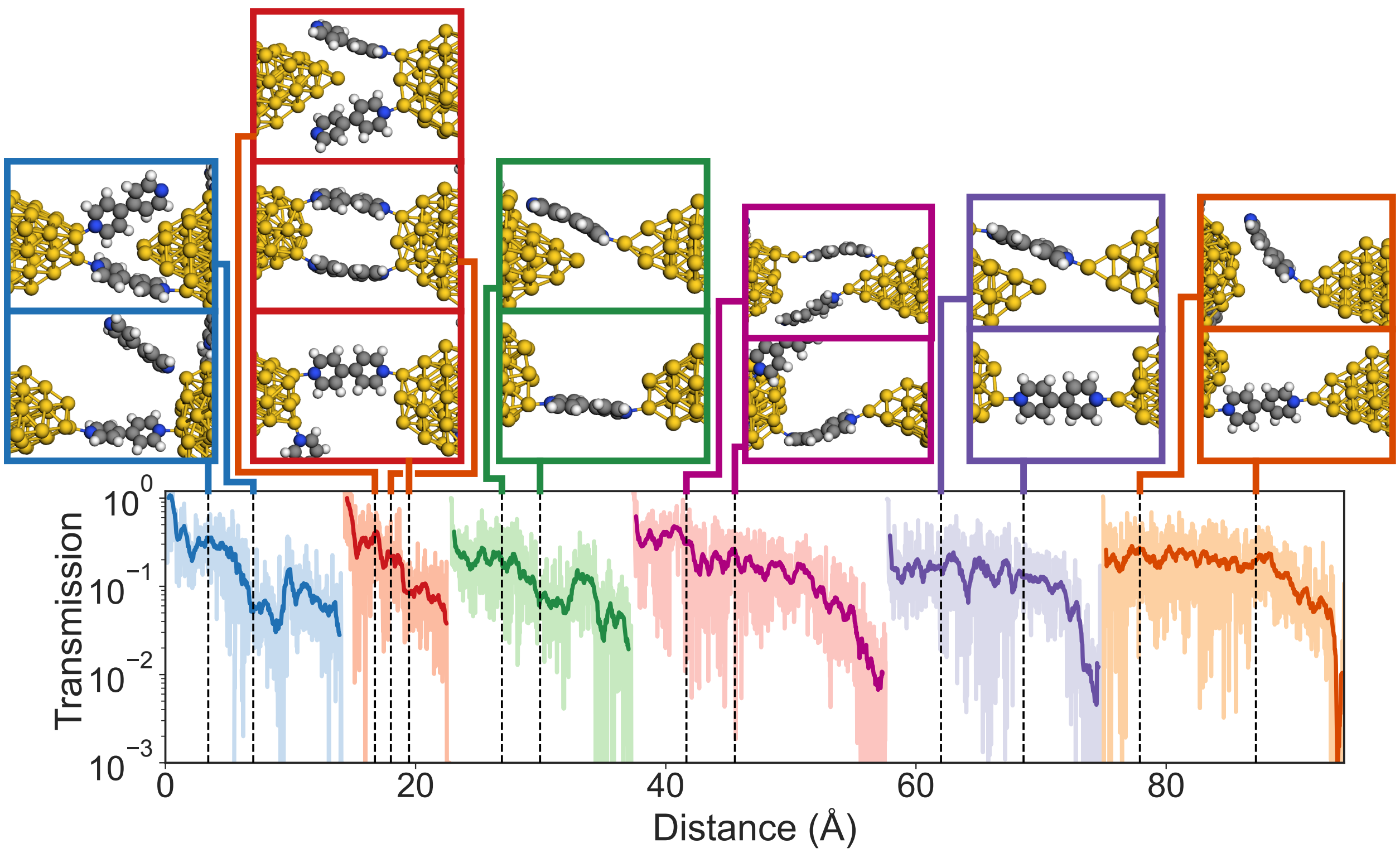

Figure S4: Visualization of the junction evolution and its impact on the conductance. (top) Snapshots from the MD trajectory, stretched at a rate of 5×10^{-7} Å/fs. (bottom) Transmission traces from the MD trajectory, where dashed lines indicate the configurations captured in the top snapshots. To show the general evolution of the transmission, we plot the actual transmission (shown in lighter hues) and its smoothed average (shown in darker hues).

Correlation with gold metrics

Figure S5 compares the transmission from selected finetuned NEP trajectories with several descriptors of the Au junction geometry (see figure caption for a description of the metrics). No single descriptor shows a clear one-to-one correspondence with the transmission. In the two-molecule traces, there are some transmission changes that coincide with abrupt changes in multiple descriptors (black dashed circles), whereas the one-molecule traces exhibit fluctuations of similar magnitude in the Au metrics without comparably abrupt transmission changes. This suggests that the high-conductance state is not controlled by a single geometric variable, but rather by a complex junction configuration involving both the molecules and the Au contact structure.

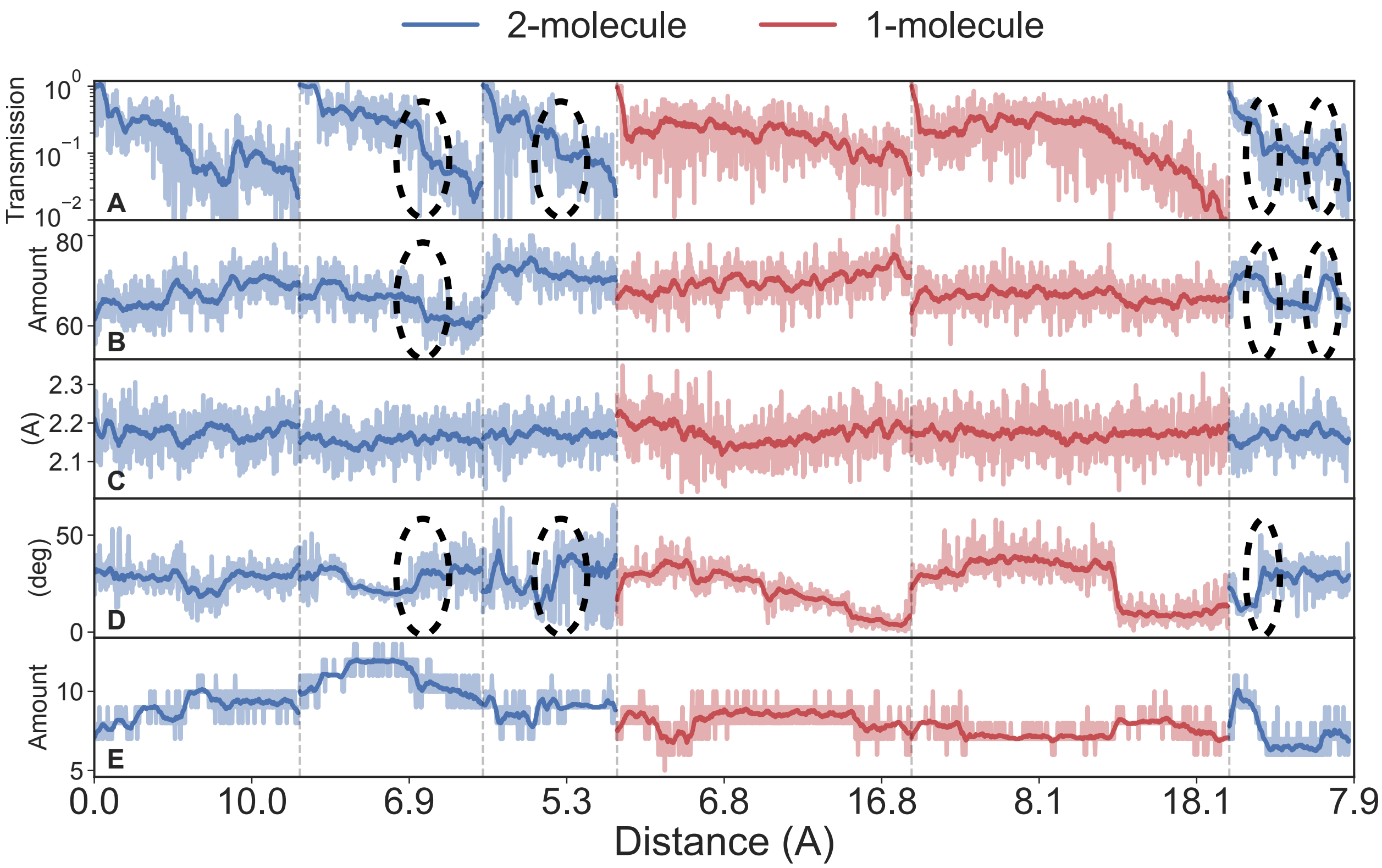

Figure S5: Transmission plotted against different metrics related to the gold electrodes. (A) Transmission for a few samples from the finetuned NEP trajectories. (B) Number of undercoordinated Au atoms there are in the gap region. 'Undercoordinated' is defined as Au atoms with fewer than 8 atoms close by where 'close' means less than 3 Å. (C) Smallest nearest distance from any N to the upper electrode. (D) Mean tilt angle of the mid-gap molecules, defined from the N-N axis relative to the z axis. Lower values, more upright molecule. (E) Number of N atoms whose nearest upper-electrode Au atom lies within 3 A. The blue color denotes trajectories that have two molecules in the junction and red trajectories with a single molecule. Trajectory labels were assigned based on visual inspection. Both the transmission and the corresponding metrics were smoothed with a moving average. The black, dashed circles denote abrupt changes in transmission and corresponding drops/rises in gold metrics.

Gibbs free energy of two 4,4'-bipyridines

We calculated the enthalpy and Gibbs free energy changes for the reaction detailed in Scheme S1, using settings similar to those in "Energy of two 4,4'-bipyridines under an electrostatic field". Additionally, we performed the calculations with the following changes in settings: we use the M06-2X functional[S6] with only Grimme's D3 dispersion correction (no BJ damping). We use the def2-TZVPPD basis set. For the single 44BPY, we set the grid

to *m4* and converge the maximum norm of the Cartesian gradient up to 10^{-4}. Because the supermolecule has very soft modes, we set the grid to *m5*, set the SCF convergence threshold to 10^{-10}, and converge the maximum norm of the Cartesian gradient up to 10^{-7}. The supermolecule has three low-lying negative frequencies. These soft modes of noncovalently bound molecules are known to be challenging to estimate accurately.[S7,S8] The resulting harmonic thermodynamic estimates should therefore be interpreted with caution, especially for the entropy. Furthermore, we perform a counterpoise correction to the electronic energy of the supermolecule to estimate the basis set superposition error.[S9]

A functional in the same family as M06-2X, the M05-2X functional, potentially overestimates the Gibbs free energy though there are few large benchmarks on Gibbs free energy of noncovalently bonded supermolecules.[S7,S8] We assume the same systematic error is present in the M06-2X functional.

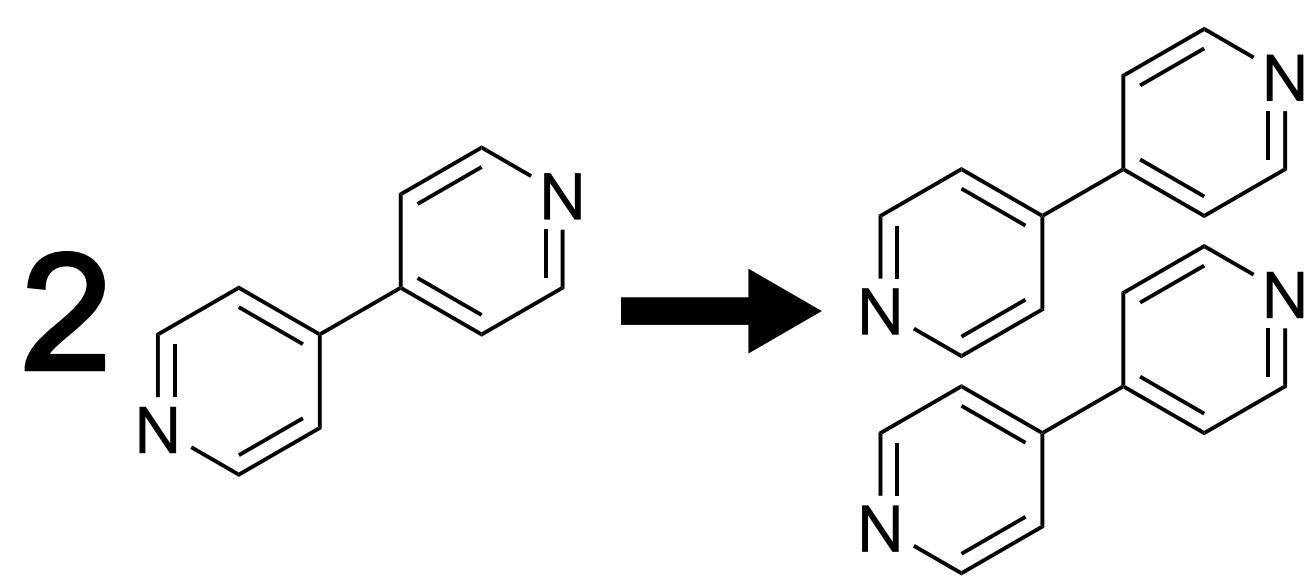

Scheme S1: Two 4,4'-bipyridines π-stacking.

Table S2 lists the electronic energy, the enthalpy, and the Gibbs free energy for a single 44BPY (G(44BPY)), for two π-stacked 44BPYs (G(44BPY$_{stacked}$)). The enthalpy and the Gibbs free energy are both listed with the zero point vibrational energy included. The resulting change in Gibbs free energy as defined by

$$\Delta \mathrm{G} = \mathrm{G}(44BPY_{stacked}) - 2 \times \mathrm{G}(44BPY) \tag{2}$$

and the change in enthalpy is similarly defined as

$$\Delta \mathrm{H} = \mathrm{H}(44BPY_{stacked}) - 2 \times \mathrm{H}(44BPY). \tag{3}$$

Table S2: Energies at 298K for the reaction in Scheme 1.

M06-2X-D3(0)	Single 44BPY	Supermolecule
Electronic[a,b]	-1300556.5	-2601147.6
Enthalpy[a]	437.77	872.98
Gibbs free energy[a]	322.26	709.64

[a] kJ/mol.
[b] The energy of the supermolecule is counterpoise corrected.

Using Equation 2 and Equation 3, we get the following changes in Gibbs free energy and enthalpy:

$$\Delta H = -37.23 \text{ kJ/mol} \tag{4}$$

$$\Delta G = 30.45 \text{ kJ/mol.} \tag{5}$$

Although the binding energy of the two 44BPYs is large (ΔH is large), their aggregation in the gas phase does not happen spontaneously because of the unfavorable change in entropy. However, within a junction, the scenario is different. Here, a molecule is already positioned between the two electrodes, which makes the entropy change between the unbound and bound states more favorable, thus reducing ΔG and facilitating spontaneous binding.

1D-histogram of transmission traces

Figure S6 shows 1D-histograms of traces with a single molecule (green) or two molecules (red) bridging the electrodes. Each histogram is constructed from three samples. With so few samples, neither histogram resolves two distinct conductance states. For ease of comparison and because we include one more trace in each data set than shown in the main manuscript, we also illustrate the traces in Figure S7. Note that the dashed traces have

already been shown in the main manuscript in Figure 2.

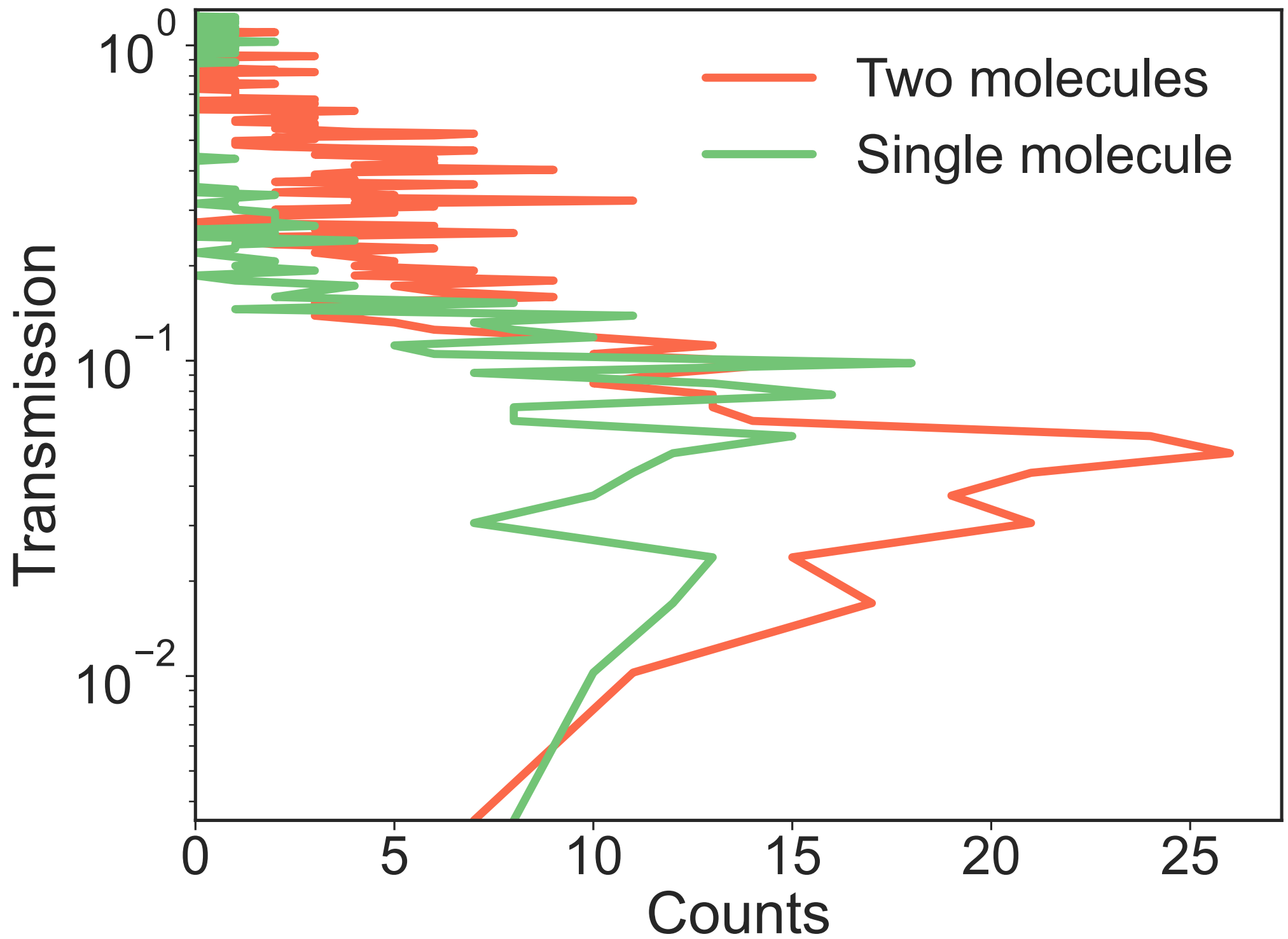

Figure S6: 1D-histograms of the transmission of traces with a single molecule (green) or two molecules (red) bridging the electrodes. Note that each histogram is constructed from three samples.

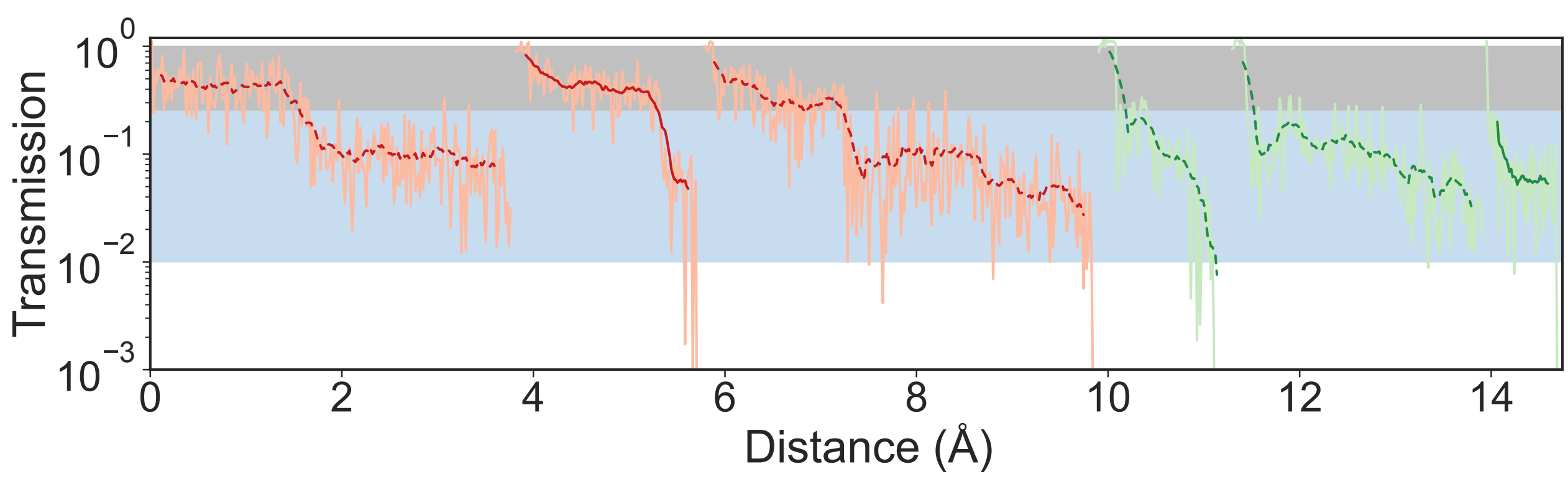

Figure S7: Transmission traces from the MD trajectories. To show the general evolution of the transmission, we plot the actual transmission (shown in lighter hues) and its smoothed average (shown in darker hues). Traces are displaced along the x-axis for clarity. The grey and blue background in the plot serve as visual aids to delineate the two conductance states. Note that the dashed traces have already been shown in the main manuscript in Figure 2.

Ab initio MD

To further validate our results and assess the possibility of two 44BPYs forming a stable supramolecular complex within the junction, we performed short *ab initio* MD trajectories. These simulations were carried out using the SIESTA code. As a first step, we performed geometry optimization with constrained atomic positions. The top gold layer and the bottom three gold layers were fixed (see Figure S8). The structure was relaxed using the Broyden method for up to 512 steps, with a force convergence threshold of 0.08 eV/Å. A double-zeta plus polarization (DZP) basis set was used for all atoms except Au, for which a single-zeta (SZ) basis was applied. The PBE exchange-correlation functional was used with DFT-D3 dispersion corrections enabled. A $1 \times 1 \times 1$ Monkhorst-Pack k-point mesh was applied.

Following relaxation, we initiated the MD trajectory using the optimized structure. The same atomic constraints were maintained. The simulation was run in the NVE ensemble with an initial temperature of 300 K. We used a time step of 0.5 fs. The junction with two 44BPYs was run for 4.7565 ps and the junction with a single 44BPY was run for 3.446 ps. Au atoms were described with a SZ basis, and the 44BPYs were described with DZP. All other computational parameters were kept the same as the relaxation step.

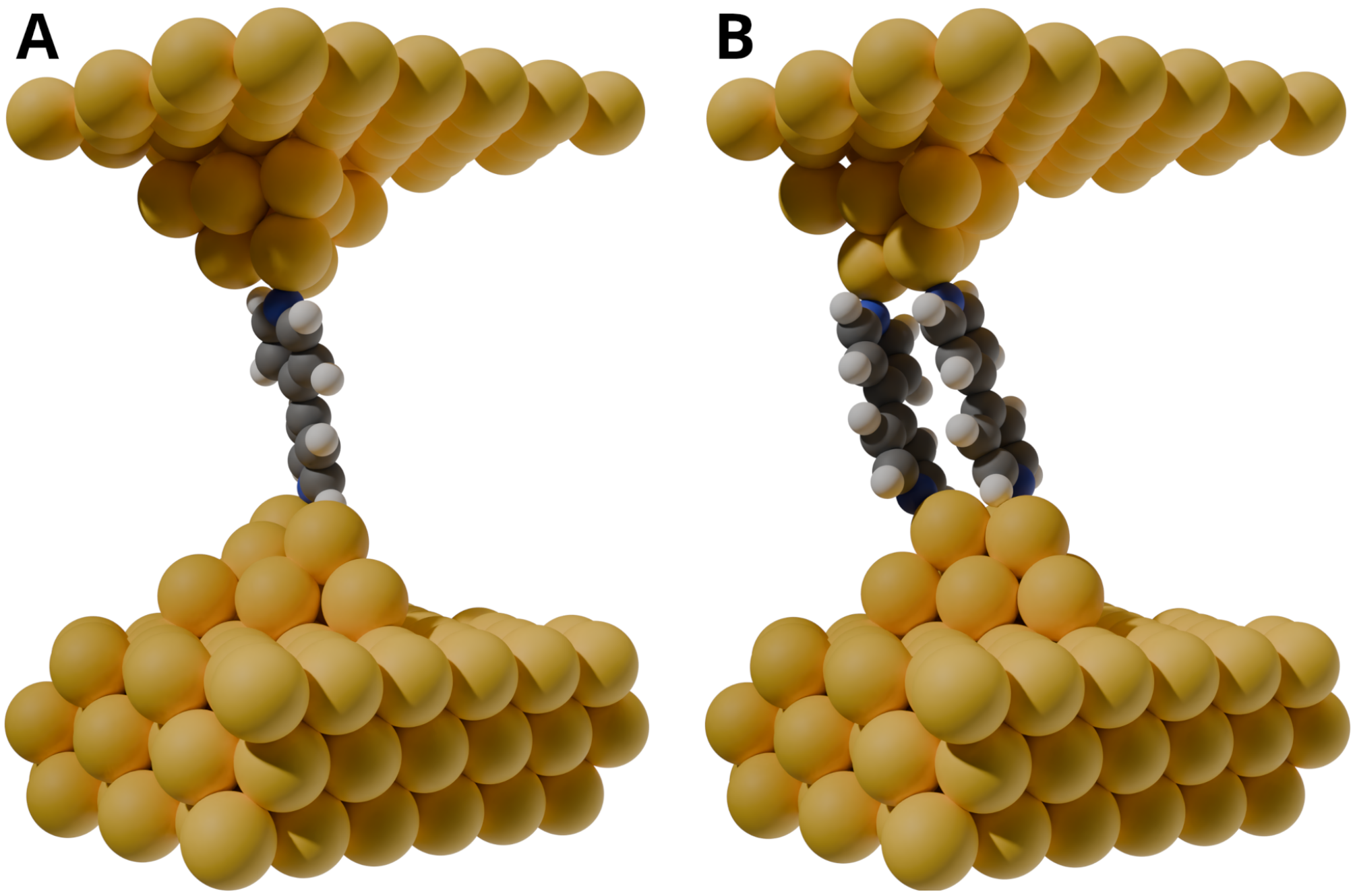

Figure S8: Example structures from the *ab initio* MD trajectories. (A) Junction with a single 44BPY. (B) Junction with two 44BPYs.

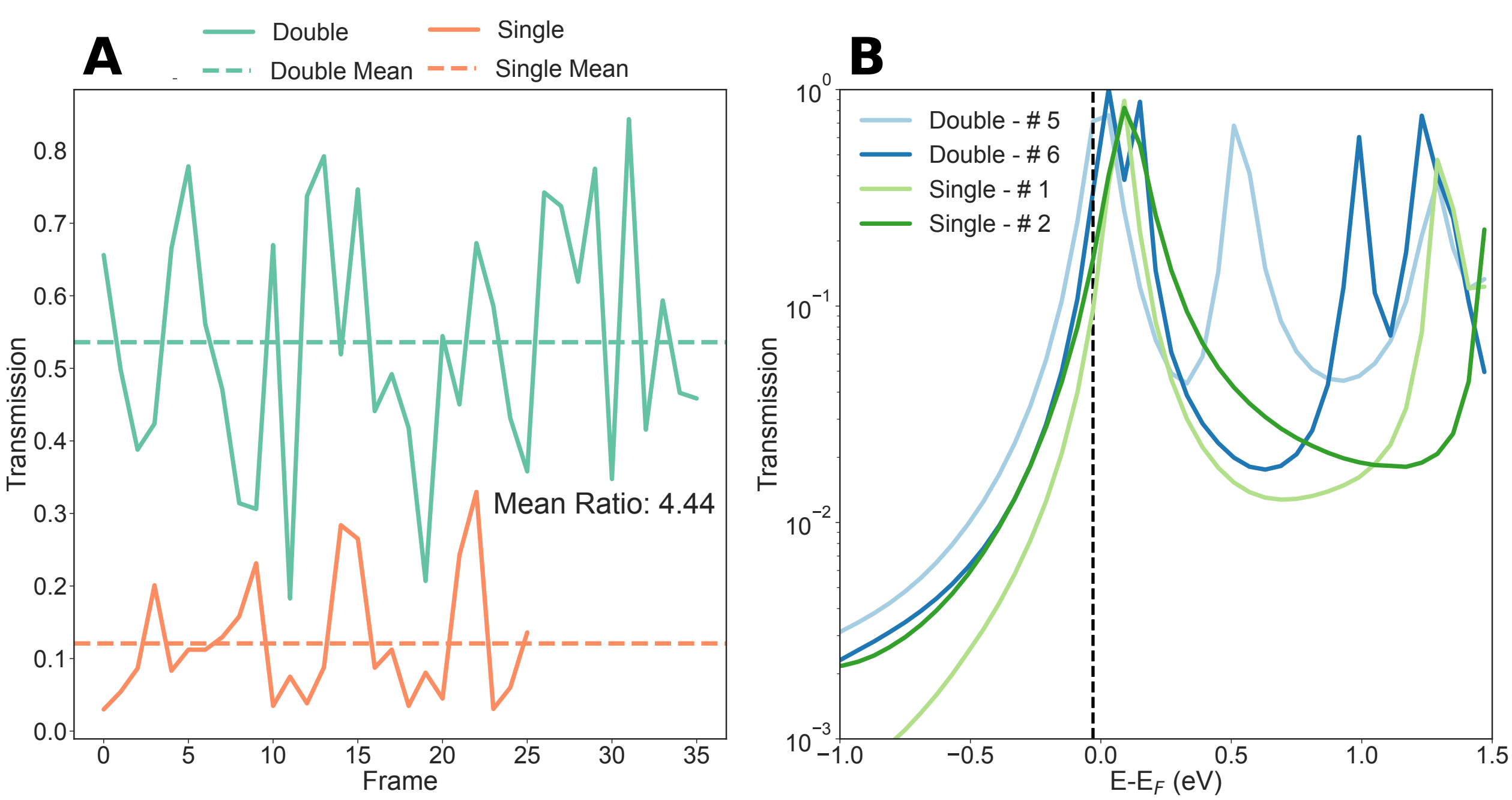

Figure S9: Electronic transmission of *ab initio* MD trajectories. (A) Electronic transmission for junctions with two (green solid line) and one (orange solid line) 44BPY molecules. Dotted lines show the mean transmission across sampled frames. The mean ratio shown is the ratio of the average transmission from the two-44BPY junction to that of the single-44BPY junction, calculated using Equation 6.

We show the transmission calculated from uniformly sampled frames of each trajectory in Figure S9A. The ratio between the average transmission of junctions containing two 44BPY molecules (green dotted line) and those with one 44BPY molecule (orange dotted line) is computed as:

$$m_{double}/m_{single}. \tag{6}$$

The mean ratio is 4.44, well above 2, indicating that the two-molecule junction conducts more strongly than expected from a simple doubling of an otherwise unchanged single-molecule conductance

Individual transmission traces are shown in Figure S9B. The transmission is mediated by the lowest unoccupied molecular orbital (LUMO), consistent with previous experimental and theoretical studies.[S10,S11] However, it is unlikely that the LUMO resonance would lie this close to the Fermi level under experimental conditions. Consequently, the calculated transmission is higher than experimental values. Nevertheless, we expect that applying a scissor correction[S12] or using a functional without the known shortcomings of PBE would still yield a transmission ratio significantly greater than 2.

Hückel modelling of two 4,4'-bipyridines

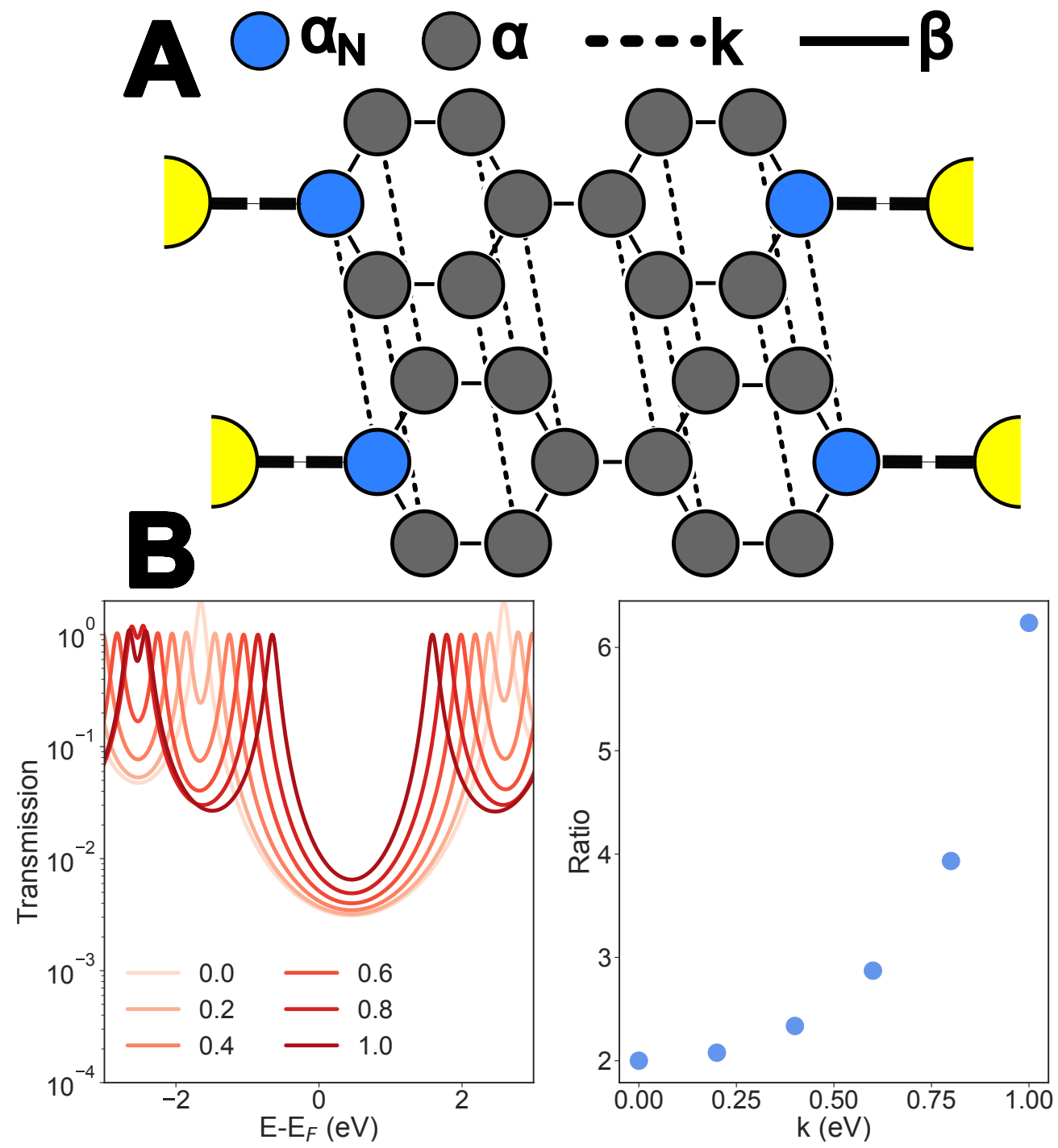

Figure S10: Electronic transmission for two interacting 44BPY molecules. (A) Hückel model of two 44BPYs with increasing interaction. Blue circles represent nitrogens, grey circles represent carbons, dotted lines indicate weak interactions, solid lines indicate nearest-neighbor interactions, and yellow half-circles represent gold electrodes. Dashed lines between the nitrogens and the electrodes depict the electrode-molecule coupling. (B) Left: Electronic transmission for increasing values of the intermolecular coupling parameter k. The legend gives the value of k and the darker red corresponds to higher k. Right: Transmission ratio between the single-molecule and the two-molecule configurations as a function of k.

As a simple illustrative model, we used Hückel theory combined with non-equilibrium Green's function (NEGF) transport to examine how weak intermolecular coupling between two 44BPY molecules can affect transmission. Although the findings from the main manuscript suggest that direct intermolecular coupling is not the full explanation for the enhanced conductance, this model remains useful for showing that even modest coupling between two molecules can substantially increase transmission

Figure S10A shows our modelling approach, where two 44BPY molecules interact through weak intermolecular couplings. Nitrogen atoms are represented by blue circles (α_N) and carbon atoms by grey circles (α), with solid lines illustrating nearest neighbor interactions

(β), and dashed lines illustrating interactions between corresponding sites on the two 44BPY (k). In Equation 7, we show an example of a Hückel matrix for a single 44BPY. In our model, we set $\alpha = 0$ eV, $\beta = -3$ eV, with k ranging from 0.0 to 1.0 eV, adopting $\alpha_N = \beta/2$ eV based on values for pyridine.[S13] Coupling to the electrodes is set to 0.5 eV. We couple both molecules to both electrodes on either end (depicted by yellow half-circles in Figure S10A) through the nitrogens.

The left plot of Figure S10 shows that increasing the intermolecular coupling, raises the transmission at the Fermi level. This increase is primarily because the gap between the highest occupied molecular orbital (HOMO) and the LUMO narrows.

Plotting the ratio between the transmission of a single 44BPY and when two 44BPYs increasingly interact (right plot of Figure S10B), we see that the conductance of two molecules could be many times higher than the single-molecule case – over six times higher at $k = 1.0$. While we do not expect this ratio to be exactly reproduced in an experiment, it underscores the substantial impact that even small interactions can have on conductance. Such interactions lead to a much greater increase in conductance than would be expected if the molecules were behaving as two parallel, non-interacting wires.

Hückel matrix of a single 4,4'-bipyridine

We show only the Hückel matrix for a single 44BPY (Equation 7), because the corresponding matrix for two interacting 44BPYs is considerably larger. Figure S11 shows the atom numbering used to define the sites in the Hückel matrix.

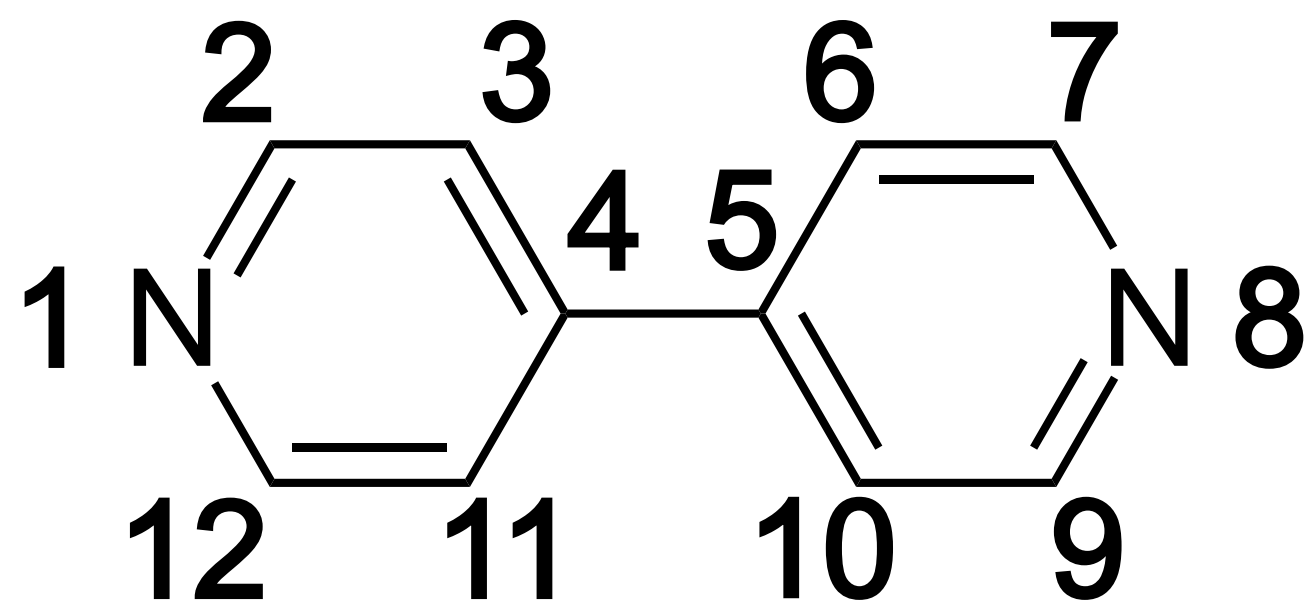

Figure S11: 44BPY numbered according to the sites in the Hückel matrix shown in Equation 7.

	1	2	3	4	5	6	7	8	9	10	11	12
1	N_α	β	0	0	0	0	0	0	0	0	0	β
2	β	α	β	0	0	0	0	0	0	0	0	0
3	0	β	α	β	0	0	0	0	0	0	0	0
4	0	0	β	α	β	0	0	0	0	0	β	0
5	0	0	0	β	α	β	0	0	0	β	0	0
6	0	0	0	0	β	α	β	0	0	0	0	0
7	0	0	0	0	0	β	α	β	0	0	0	0
8	0	0	0	0	0	0	β	N_α	β	0	0	0
9	0	0	0	0	0	0	0	β	α	β	0	0
10	0	0	0	0	β	0	0	0	β	α	0	0
11	0	0	0	β	0	0	0	0	0	0	α	β
12	β	0	0	0	0	0	0	0	0	0	β	α

(7)

Energy of two 4,4'-bipyridines under an electrostatic field

In the following, we examine how an applied electrostatic field affects the energy profile of two interacting 44BPY molecules along their intermolecular separation coordinate as was observed in a cryogenic mechanically controlled break junction setup.[S14]

We performed calculations with TURBOMOLE 7.6[S15] using the B3LYP functional with Grimme's D3 dispersion correction and Becke-Johnson (BJ) damping.[S16,S17] We use the

'resolution of identity' approximation and the def2-SVP basis set for all atoms.[S18] We use an SCF convergence threshold of 10^{-8}.

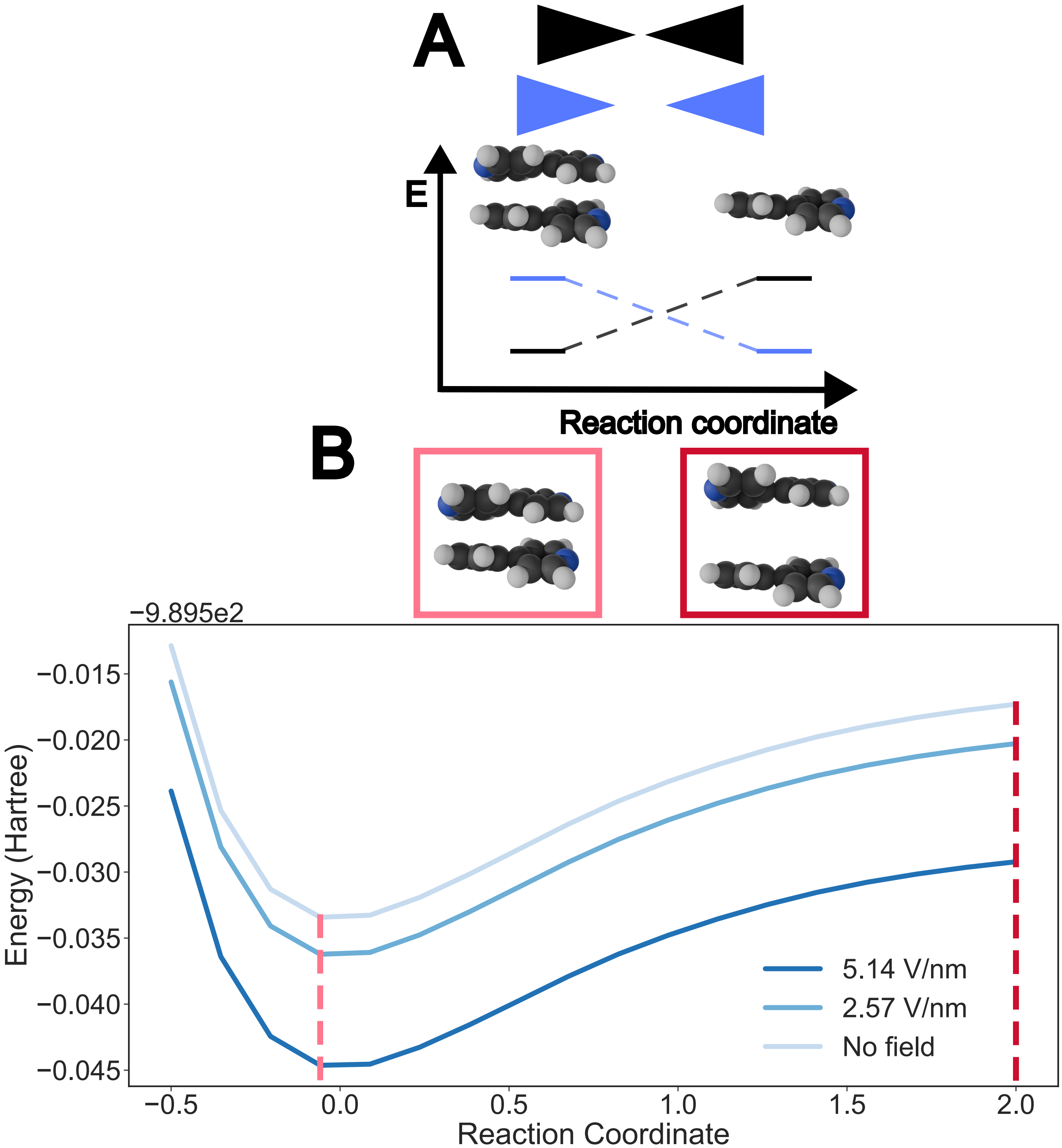

Figure S12: (A) Reaction coordinate diagram displaying energy states for a break junction with small (black triangles, lines) and large (blue triangles, lines) gaps, indicating predominant configurations as bipyridine pairs or single bipyridines respectively. (B) Energy diagram showing the impact of an applied electrostatic field along the z-axis (axis defined by the nitrogens) on bipyridine end-states, depicted in red boxes (ground state in pink, near non-interacting state in red), with dashed lines marking the energetic position of these states and increasingly blue lines indicating increasing field strength. The reaction coordinate represents the distance between bipyridines.

Figure S12A illustrates the experimental setup and resulting energy diagram. When the junction gap is small (black triangles and black lines in energy diagram), the 44BPY-dimer is energetically preferred. This configuration is consistent with our molecular dynamics results in the main manuscript, which show that without closely positioned gold electrodes, the gap becomes too large to accommodate the dimer.

When the junction gap is expanded (blue triangles and blue lines in energy diagram), the single-molecule configuration becomes energetically preferred because the gap can no longer accommodate two 44BPY molecules.

Figure S12B illustrates how applying an electrostatic field affects two interacting 44BPY molecules. We applied the field along the axis defined by the two nitrogens in a single 44BPY molecule. Starting from the ground state where two 44BPY molecules interact (light red box), we vary the intermolecular distance along the reaction coordinate. In the red box, we show the 44BPY-dimer at the largest intermolecular distance considered.

In the plot at the bottom of Figure S12B, we show how the energy of the dimer system changes along the reaction coordinate – that is, with increasing distance between the two 44BPYs. We show this for three different field strengths: no field in grey, a field strength of 2.57 V/nm in light blue, and a field strength of 5.14 V/nm in dark blue. The dashed lines indicate the energetic position of the two configurations shown in the light red and red boxes.

As the field strength increases, the energy decreases across the whole reaction coordinate, including both the close-contact configuration and the large-separation limit where the two 44BPY molecules are effectively noninteracting. The calculations suggest that the applied field stabilizes the two-molecule manifold as a whole. If states with two molecules in the junction are only marginally unfavorable in the absence of a field, such a lowering could increase their statistical weight relative to lower-occupancy junction states. Because our model does not explicitly include the corresponding one-molecule junction configuration, molecule exchange, or the electrodes, this interpretation is necessarily qualitative.

DFT modelling of single configurations

To further assess the validity of the DFT results, we also calculate the electronic transmission using FHI-aims.[S19,S20] Electronic transmission was calculated within the NEGF framework using geometries optimized with the PBE exchange-correlation functional. All-electron numeric atom-centered basis sets with tight computational settings were employed. Total energies and electronic structures were obtained from these optimized geometries and used as input for the transport calculations.

To assess the structural origin of the enhanced transmission, we analyzed several configurations derived from the relaxed two-44BPY junction. Specifically, we compared the transmission from the fully relaxed two-molecule structure double 44BPY (red line in Figure S13) with that from structures where one 44BPY molecule was removed. In the 'Unrelax' cases, one molecule was removed without further structural relaxation. In contrast, the 'Relax' cases involved relaxing the resulting single-molecule junction after removal of the second molecule. This comparison allows us to distinguish the effect of structural rearrangement from simple molecule removal on the resulting transmission.

We evaluated the transmission at the Fermi level and at -1 eV, as previous experiments have suggested that the Fermi level lies approximately 1 eV below the LUMO of 44BPY.[S11] The resulting ratios between the double- and single-molecule junctions are summarized in Table S3.

At the Fermi level, the ratio exceeds 30, while at -1 eV it is slightly above 2. These results demonstrate that the enhancement in transmission for the two-molecule junction can be substantial, particularly near the Fermi level.

Table S3: Ratios of transmission

Energy	Relaxed	Unrelaxed
Fermi level (0 eV)	33.23	48.19
Shift to -1 eV	3.189	2.273

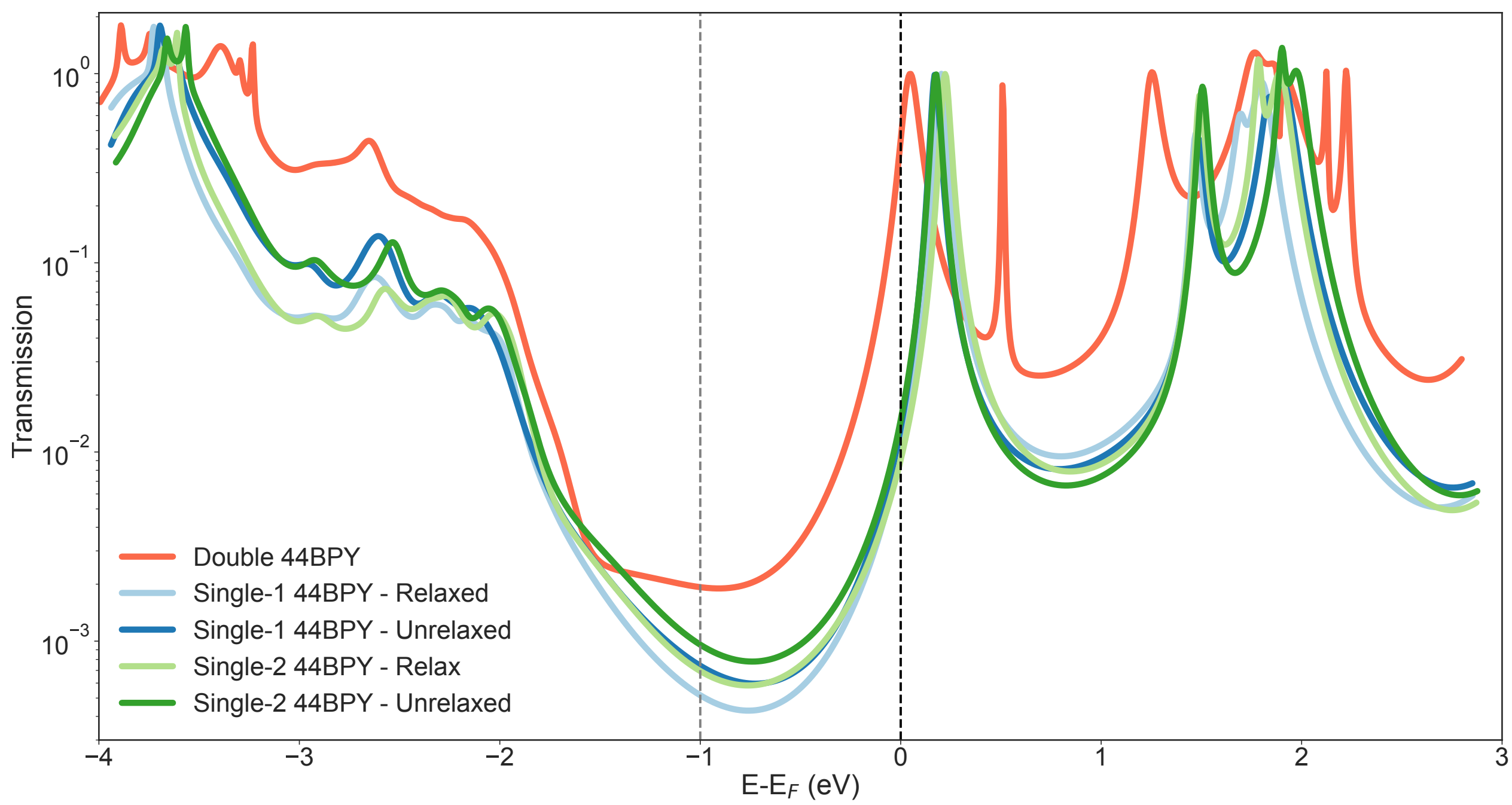

Figure S13: Electronic transmission for the two-44BPY junction and derived single-44BPY configurations. The red curve shows the original relaxed two-44BPY junction. Blue and dark blue correspond to removal of molecule 1 with and without relaxation, respectively. Light green and dark green show the analogous cases for molecule 2.

Experimental high- and low-conductance values

Table S4 lists experimental conductance values for 44BPY experiments.

Table S4: Experimental conductance values

Reference	High (G_0)	Low (G_0)	Experimental setup
Kim et al.[S11]	$\approx 4 \times 10^{-4}$	$\approx 1 \times 10^{-4}$	STM-BJ
Quek et al.[S21]	6×10^{-4}	1.6×10^{-4}	STM-BJ
Baghernejad et al.[S22]	$10^{-3.3}$	$10^{-2.9}$	STM-BJ
Makk et al.[S23]	$\approx 1 \times 10^{-3}$	2×10^{-4}	STM-BJ
Long et al.[S24]	$\approx 10^{-3}$	$\approx 10^{-3.8}$	STM-BJ

References

(S1) Schaul, T.; Glasmachers, T.; Schmidhuber, J. High dimensions and heavy tails for natural evolution strategies. Proceedings of the 13th Annual Conference on Genetic and Evolutionary Computation. New York, NY, USA, 2011; p 845852.

(S2) Bro-Jørgensen, W.; Hamill, J. M.; Donadio, D.; Solomon, G. C. Bridging the Gap: Using Machine Learning Force Fields to Simulate Gold Break Junctions at Pulling Speeds Closer to Experiments. *ACS Nano* **2025**, *19*, 39735–39746.

(S3) Zhang, H.; Juraskova, V.; Duarte, F. Modelling chemical processes in explicit solvents with machine learning potentials. *Nature Communications* **2024**, *15*, 6114.

(S4) Himanen, L.; Jäger, M. O. J.; Morooka, E. V.; Federici Canova, F.; Ranawat, Y. S.; Gao, D. Z.; Rinke, P.; Foster, A. S. DScribe: Library of descriptors for machine learning in materials science. *Computer Physics Communications* **2020**, *247*, 106949.

(S5) Laakso, J.; Himanen, L.; Homm, H.; Morooka, E. V.; Jäger, M. O.; Todorović, M.; Rinke, P. Updates to the DScribe library: New descriptors and derivatives. *The Journal of Chemical Physics* **2023**, *158*.

(S6) Zhao, Y.; Truhlar, D. G. The M06 suite of density functionals for main group thermochemistry, thermochemical kinetics, noncovalent interactions, excited states, and transition elements: two new functionals and systematic testing of four M06-class functionals and 12 other functionals. *Theoretical Chemistry Accounts* **2008**, *120*, 215–241.

(S7) Michalkova, A.; Gorb, L.; Hill, F.; Leszczynski, J. Can the Gibbs Free Energy of Adsorption Be Predicted Efficiently and Accurately: An M05-2X DFT Study. *The Journal of Physical Chemistry A* **2011**, *115*, 2423–2430.

(S8) Scott, A. M.; Gorb, L.; Burns, E. A.; Yashkin, S. N.; Hill, F. C.; Leszczynski, J. Toward Accurate and Efficient Predictions of Entropy and Gibbs Free Energy of Adsorption

of High Nitrogen Compounds on Carbonaceous Materials. *The Journal of Physical Chemistry C* **2014**, *118*, 4774–4783.

(S9) Boys, S.; Bernardi, F. The calculation of small molecular interactions by the differences of separate total energies. Some procedures with reduced errors. *Molecular Physics* **1970**, *19*, 553–566.

(S10) Kamenetska, M.; Quek, S. Y.; Whalley, A. C.; Steigerwald, M. L.; Choi, H. J.; Louie, S. G.; Nuckolls, C.; Hybertsen, M. S.; Neaton, J. B.; Venkataraman, L. Conductance and Geometry of Pyridine-Linked Single-Molecule Junctions. *Journal of the American Chemical Society* **2010**, *132*, 6817–6821.

(S11) Kim, T.; Darancet, P.; Widawsky, J. R.; Kotiuga, M.; Quek, S. Y.; Neaton, J. B.; Venkataraman, L. Determination of Energy Level Alignment and Coupling Strength in 4,4-Bipyridine Single-Molecule Junctions. *Nano Letters* **2014**, *14*, 794–798.

(S12) Quek, S. Y.; Venkataraman, L.; Choi, H. J.; Louie, S. G.; Hybertsen, M. S.; Neaton, J. B. AmineGold Linked Single-Molecule Circuits: Experiment and Theory. *Nano Letters* **2007**, *7*, 3477–3482.

(S13) Purcell, W. P.; Singer, J. A. A brief review and table of semiempirical parameters used in the Hueckel molecular orbital method. *Journal of Chemical & Engineering Data* **1967**, *12*, 235–246.

(S14) Mezei, G.; Balogh, Z.; Magyarkuti, A.; Halbritter, A. Voltage-Controlled Binary Conductance Switching in Gold–4,4'-Bipyridine–Gold Single-Molecule Nanowires. *The Journal of Physical Chemistry Letters* **2020**, *11*, 8053–8059.

(S15) TURBOMOLE V7.6 2020, a development of University of Karlsruhe and Forschungszentrum Karlsruhe GmbH, 1989-2007, TURBOMOLE GmbH, since 2007; available from
`https://www.turbomole.org`.

(S16) Grimme, S.; Ehrlich, S.; Goerigk, L. Effect of the damping function in dispersion corrected density functional theory. *Journal of Computational Chemistry* **2011**, *32*, 1456–1465.

(S17) Smith, D. G. A.; Burns, L. A.; Patkowski, K.; Sherrill, C. D. Revised Damping Parameters for the D3 Dispersion Correction to Density Functional Theory. *The Journal of Physical Chemistry Letters* **2016**, *7*, 2197–2203.

(S18) Weigend, F.; Ahlrichs, R. Balanced basis sets of split valence, triple zeta valence and quadruple zeta valence quality for H to Rn: Design and assessment of accuracy. *Phys. Chem. Chem. Phys.* **2005**, *7*, 3297.

(S19) Blum, V.; Gehrke, R.; Hanke, F.; Havu, P.; Havu, V.; Ren, X.; Reuter, K.; Scheffler, M. Ab initio molecular simulations with numeric atom-centered orbitals. *Computer Physics Communications* **2009**, *180*, 2175–2196.

(S20) Havu, V.; Blum, V.; Havu, P.; Scheffler, M. Efficient O(N) integration for all-electron electronic structure calculation using numeric basis functions. *Journal of Computational Physics* **2009**, *228*, 8367–8379.

(S21) Quek, S. Y.; Kamenetska, M.; Steigerwald, M. L.; Choi, H. J.; Louie, S. G.; Hybertsen, M. S.; Neaton, J. B.; Venkataraman, L. Mechanically controlled binary conductance switching of a single-molecule junction. *Nature Nanotechnology* **2009**, *4*, 230–234.

(S22) Baghernejad, M.; Manrique, D. Z.; Li, C.; Pope, T.; Zhumaev, U.; Pobelov, I.; Moreno-García, P.; Kaliginedi, V.; Huang, C.; Hong, W.; Lambert, C.; Wandlowski, T. Highly-effective gating of single-molecule junctions: an electrochemical approach. *Chem. Commun.* **2014**, *50*, 15975–15978.

(S23) Makk, P.; Tomaszewski, D.; Martinek, J.; Balogh, Z.; Csonka, S.; Wawrzyniak, M.;

Frei, M.; Venkataraman, L.; Halbritter, A. Correlation Analysis of Atomic and Single-Molecule Junction Conductance. *ACS Nano* **2012**, *6*, 3411–3423.

(S24) Long, X.; Xu, W.; Duan, T.; Lin, L.; Guo, Y.; Yan, X.; Cao, J.; Hu, Y. Tuning charge transport by manipulating concentration dependent single-molecule absorption configurations. *iScience* **2024**, *27*.
